# 6-Arylpyrido[2,3-*d*]pyrimidines as Novel ATP-Competitive Inhibitors of Bacterial D-Alanine:D-Alanine Ligase

**DOI:** 10.1371/journal.pone.0039922

**Published:** 2012-08-02

**Authors:** Veronika Škedelj, Emilija Arsovska, Tihomir Tomašić, Ana Kroflič, Vesna Hodnik, Martina Hrast, Marija Bešter-Rogač, Gregor Anderluh, Stanislav Gobec, Julieanne Bostock, Ian Chopra, Alex J. O'Neill, Christopher Randall, Anamarija Zega

**Affiliations:** 1 Faculty of Pharmacy, University of Ljubljana, Ljubljana, Slovenia; 2 Faculty of Chemistry and Chemical Technology, University of Ljubljana, Ljubljana, Slovenia; 3 Biotechnical faculty, Infrastructural Center for Surface Plasmon Resonance, University of Ljubljana, Ljubljana, Slovenia; 4 Antimicrobial Research Centre and Instititue of Molecular & Cellular Biology, University of Leeds, Leeds, United Kingdom; Centre National de la Recherche Scientifique, France

## Abstract

**Background:**

ATP-dependent D-alanine:D-alanine ligase (Ddl) is a part of biochemical machinery involved in peptidoglycan biosynthesis, as it catalyzes the formation of the terminal D-ala-D-ala dipeptide of the peptidoglycan precursor UDPMur*N*Ac-pentapeptide. Inhibition of Ddl prevents bacterial growth, which makes this enzyme an attractive and viable target in the urgent search of novel effective antimicrobial drugs. To address the problem of a relentless increase in resistance to known antimicrobial agents we focused our attention to discovery of novel ATP-competitive inhibitors of Ddl.

**Methodology/Principal Findings:**

Encouraged by recent successful attempts to find selective ATP-competitive inhibitors of bacterial enzymes we designed, synthesized and evaluated a library of 6-arylpyrido[2,3-*d*]pyrimidine-based compounds as inhibitors of *Escherichia coli* DdlB. Inhibitor binding to the target enzyme was subsequently confirmed by surface plasmon resonance and studied with isothermal titration calorimetry. Since kinetic analysis indicated that 6-arylpyrido[2,3-*d*]pyrimidines compete with the enzyme substrate ATP, inhibitor binding to the ATP-binding site was additionally studied with docking. Some of these inhibitors were found to possess antibacterial activity against membrane-compromised and efflux pump-deficient strains of *E. coli.*

**Conclusions/Significance:**

We discovered new ATP-competitive inhibitors of DdlB, which may serve as a starting point for development of more potent inhibitors of DdlB that could include both, an ATP-competitive and D-Ala competitive moiety.

## Introduction

The worldwide emergence over the past three decades of bacterial strains resistant to most current antibiotics constitutes a serious threat to global public health and has led to considerable efforts to identify and exploit new antibacterial targets and novel antimicrobial agents [1], [2]. Bacterial cell wall peptidoglycan synthesis has proven to be a well-established and validated target for antibacterial action, since it is the site of action of the clinically important ß-lactam and glycopeptide classes of antibiotics [3]. The disaccharide-pentapeptide peptidoglycan structure is a specific and essential component of the bacterial cell wall whose main function is to preserve cell integrity by resisting high internal osmotic pressure and maintain a defined cell shape [4]. It is a common feature of both Gram-negative and Gram-positive bacteria and is therefore an attractive target for the development of broad-spectrum antibacterials [5]. The biosynthesis of peptidoglycan can be divided into 3 sequential stages, each comprising a series of reactions occurring at different locations in bacteria: in the cytoplasm (synthesis of the nucleotide precursors), on the inner side (synthesis of lipid-linked intermediates) and outer side (polymerization reactions) of the cytoplasmic membrane [6], [7]. Of the enzymes that act in the first stage, D-alanine:D-alanine ligase (Ddl) is of particular interest, as it utilizes a substrate (D-alanine), which is specific for bacterial peptidoglycan biosynthesis, and is essential for bacterial growth [8], [9]. Ddl catalyzes the ATP-dependent formation of a dipeptide D-Ala-D-Ala that subsequently occupies the terminal position of the cell wall UDP-Mur*N*Ac-pentapeptide precursor units. This terminal dipeptide plays a pivotal role in the extracellular steps of peptidoglycan assembly, where cross-linking of adjacent peptidoglycan chains occurs via breaking a dipeptide bond and forming a new one [10].

D-Alanine:D-alanine ligase (EC 6.3.2.4) was first detected in the early 1960s and soon afterwards partially purified from *Enterococcus faecalis*. Kinetic and specificity studies of the reaction were performed on the purified ligase and provided evidence for two D-Ala binding sites with different substrate affinities [11], [12]. Ddl enzymes from various Gram-negative and Gram-positive pathogens have been isolated and characterized since then, including D-alanine:D-alanine ligase from *Escherichia coli*, in which two isoforms of the enzyme exist, DdlA and DdlB, that exhibit 35% amino acid sequence identity. They also express similar kinetic characteristics, substrate specifity and sensitivity for known inhibitors, but since DdlB is more extensively studied, we focused our attention on this isoform.[7], [8].

**Figure 1:**
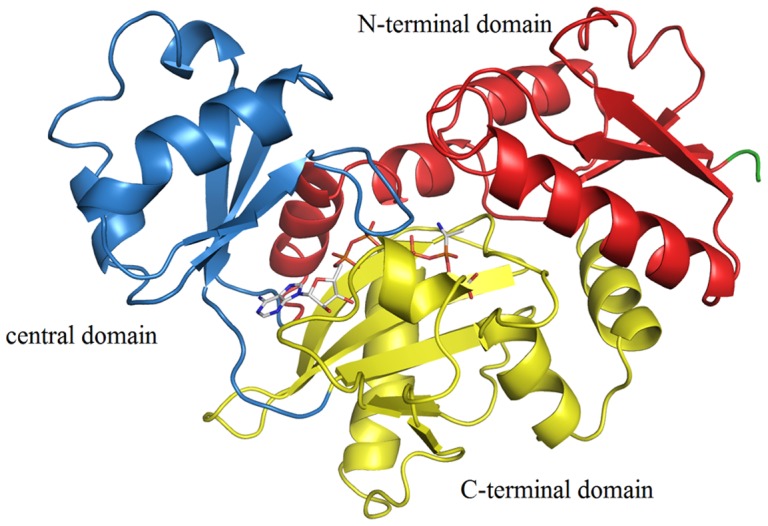
Three-dimensional structure of *E. coli* Ddl in complex with ADP (in grey sticks) and phosphoryl phosphonate-based inhibitor (PDB entry: 1IOV).

**Figure 2:**
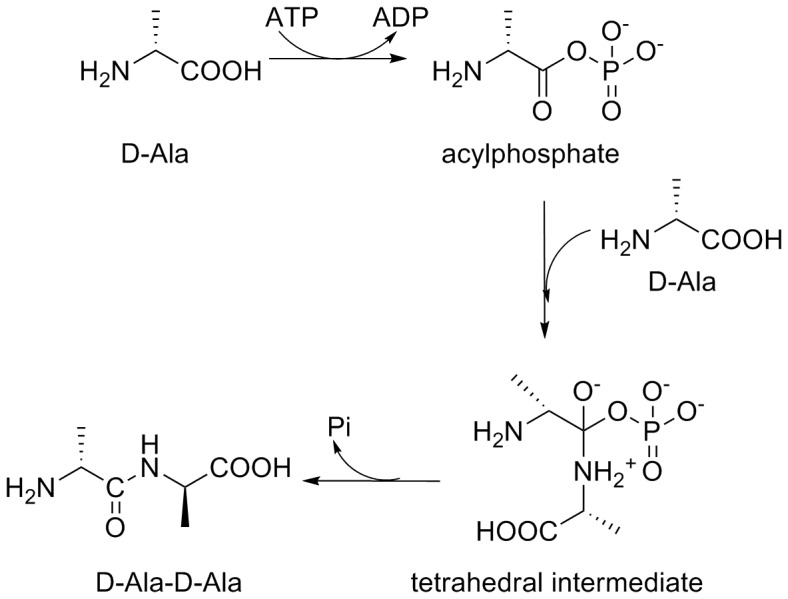
Reaction mechanism of Ddl.

DdlB consists of three α+ß domains with an unusual nucleotide-binding fold, referred to as the ATP-grasp fold [13]. The overall chemical transformation catalyzed by the ATP-grasp fold proteins is the formation of a carbon-nitrogen bond; they have, therefore, been referred to as carboxylate-amine ligases. Members of this superfamily have a common three domain molecular architecture with the nucleotide triphosphate-binding pocket positioned at the interface between the N- and C-terminal domains (Figure 1). They also have a common reaction mechanism that requires Mg^2+^ and ATP for the formation of an acylphosphate intermediate [14]. In the initial step of Ddl catalyzed reaction, ATP is bound to the free enzyme, followed by D-Ala_1_. Subsequent phosphorylation of the amino acid carboxylate by the γ-phosphate of ATP generates an acyl phosphate intermediate which is attacked by the amino group of the D-Ala_2_ to yield the dipeptide D-Ala-D-Ala (Figure 2) [8], [15], [16].

Since the first report of Ddl 50 years ago, four main categories of inhibitors of this enzyme have been described: analogues of D-Ala, analogues of product, transition state analogues and, more recently, inhibitors discovered by either screening or modeling [10]. The first discovered, and also the most important, inhibitor of Ddl is D-4-amino-3-isoxazolidone (D-cycloserine), a structural analogue of the natural substrate D-alanine with a K_i_ of 27 µM [17]. D-cycloserine is the only Ddl inhibitor that has been used in the clinic, mainly in combination with other antibiotics for the treatment of tuberculosis, but, due to its high minimal inhibitory concentration (MIC) values and neurological side effects, its use has been almost completely abandoned [18]. Since Ddl is strongly inhibited by its reaction product D-Ala-D-Ala, a wide variety of mixed dipeptide analogues have been tested for inhibition of the enzyme and several have proved to be slightly more effective inhibitors than the natural reaction product [19]. Phosphinate and phosphonate dipeptides have been described as transition-state mimetics but, despite their potent activity against isolated enzymes, they failed to show significant antibacterial activity, probably related to poor transport into bacteria [10]. Over the last few years several new inhibitor scaffolds that show no structural similarity with the substrate, product or reaction intermediate have been identified by *de novo* structure-based drug design [20], [21] and by virtual screening [22], [23], [24], [25], [26].

The lack of potent Ddl inhibitors complying with the requirements for routine use in antibacterial therapy inspired us to search for new inhibitor scaffolds for the target enzyme. Up to now most attention has been focused on substrate, product or transition-state analogues, leaving the ATP-binding site quite unexploited. Only few of existing Ddl inhibitors interfere with the binding of ATP to the target enzyme. Two flavonoids, apigenin and quercetin have proven to be potent ATP-competitive inhibitors of DdlB and *Helicobacter pylori* Ddl with antibacterial activity, but since they also act on other targets in bacteria (DNA gyrase, membrane, fatty acid biosynthesis), it is difficult to attribute their activity to the inhibition of cell wall synthesis only [22]. A common topology of the ATP-binding site of Ddl and different classes of kinases resulted in evaluation of a series of ATP competitive kinase inhibitors and identifying a few potent ATP-competitive inhibitors of *E. coli* DdlB [24]. Finally, two new and structurally diverse ATP-competitive inhibitors of DdlB from NCI database with IC_50_ values in the low micromolar concentration range were evidenced using structure-based virtual screening [25], [26].

Targeting the ATP-binding site of bacterial enzymes is associated with several problems. An ATP-competitive inhibitor of bacterial enzyme must be able to compete with the high ATP concentration in the bacterial cell (0.6–18 mM), which is similar to that in human cells (1–10 mM). Additionally, inhibitor binding to the ATP-binding site must be selective for the target bacterial enzyme over human ATP-dependent enzymes, particularly kinases. However, recent successful examples of ATP-competitive bacterial enzyme inhibitors possessing antibacterial activity and displaying good selectivity profiles with respect to human enzymes show that these challenges can be overcome [27].

Ddl belongs to the ATP-grasp superfamily which currently includes 21 groups of enzymes.[28] We studied the ATP-binding site of DdlB ligase (PDB entry: 1IOW) using ProBiS, a Web server for detecting protein binding sites based on local structural alignments, and found that the Ddl ATP-binding site is structurally similar to the those of 80 enzymes in the RCSB Protein Data Bank. Top ranked structures belong to other bacterial members of the ATP-grasp superfamily, such as Ddl from other bacterial strains, D-alanine:D-lactate ligase, carbamoyl phosphate synthetase, biotin carboxylase (BC), acetyl-CoA carboxylase and glutathione synthetase, and show less similarity to ATP-utilizing human enzymes, since only 7 ranked enzyme structures are of human origin (Table S1). Although this study included only enzymes with known crystal structure, we may assume that ATP-binding site of Ddl ligase represents a promising target for the design of ATP-competitive ligands that do not interact with human ATP-binding enzymes.

Recently, Miller et al. identified promising hits targeting the ATP-binding site of biotin carboxylase (BC) from the Pfizer series of pyridopyrimidines that emerged from a structure-based drug design program targeting eukaryotic protein kinases [29]. Based on these encouraging results and structural similarity between DdlB and BC, we developed and evaluated a library of 6-arylpyrido[2,3-*d*]pyrimidines as inhibitors of DdlB that inhibit the target enzyme with IC_50_ values in the micromolar range. Inhibitor binding was subsequently confirmed with surface plasmon resonance (SPR) and studied by isothermal titration calorimetry (ITC). Docking was performed to clarify the binding mode of the inhibitor to the DdlB ATP-binding site.

## Results and Discussion

### Design

**Figure 3:**
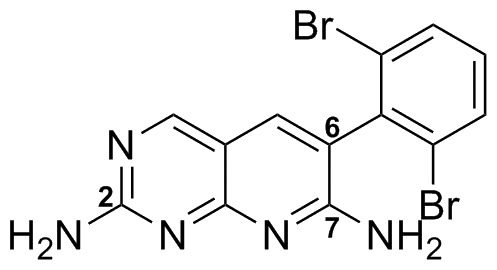
Pyridopyrimidine-based inhibitor 33 of BC based on the protein kinase inhibitor pharmacophore.

The starting point for our attempt to discover novel DdlB inhibitors was the previously described ATP-competitive BC inhibitor **33** (Figure 3), given the structural similarity between DdlB and BC ATP-binding sites. It exhibits an IC_50_ less than 5 nM for the *E. coli* BC enzyme, antibacterial activity *in vitro* and *in vivo* and a high resolution X-ray crystal structure of BC-**33** complex has been determined [29].

**Figure 4:**
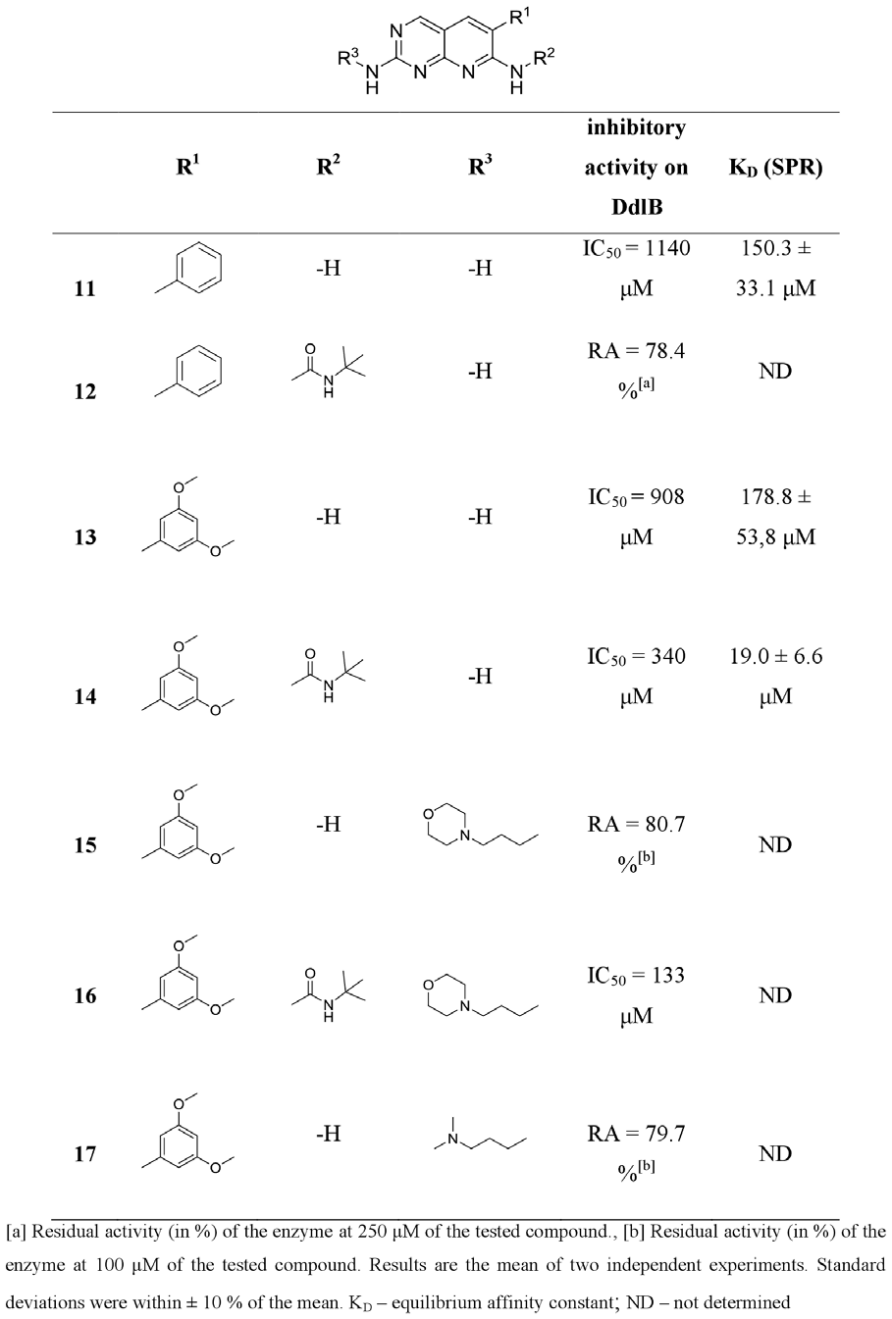
Inhibitory activities of pyridopyrimidines toward DdlB.

**Figure 5:**
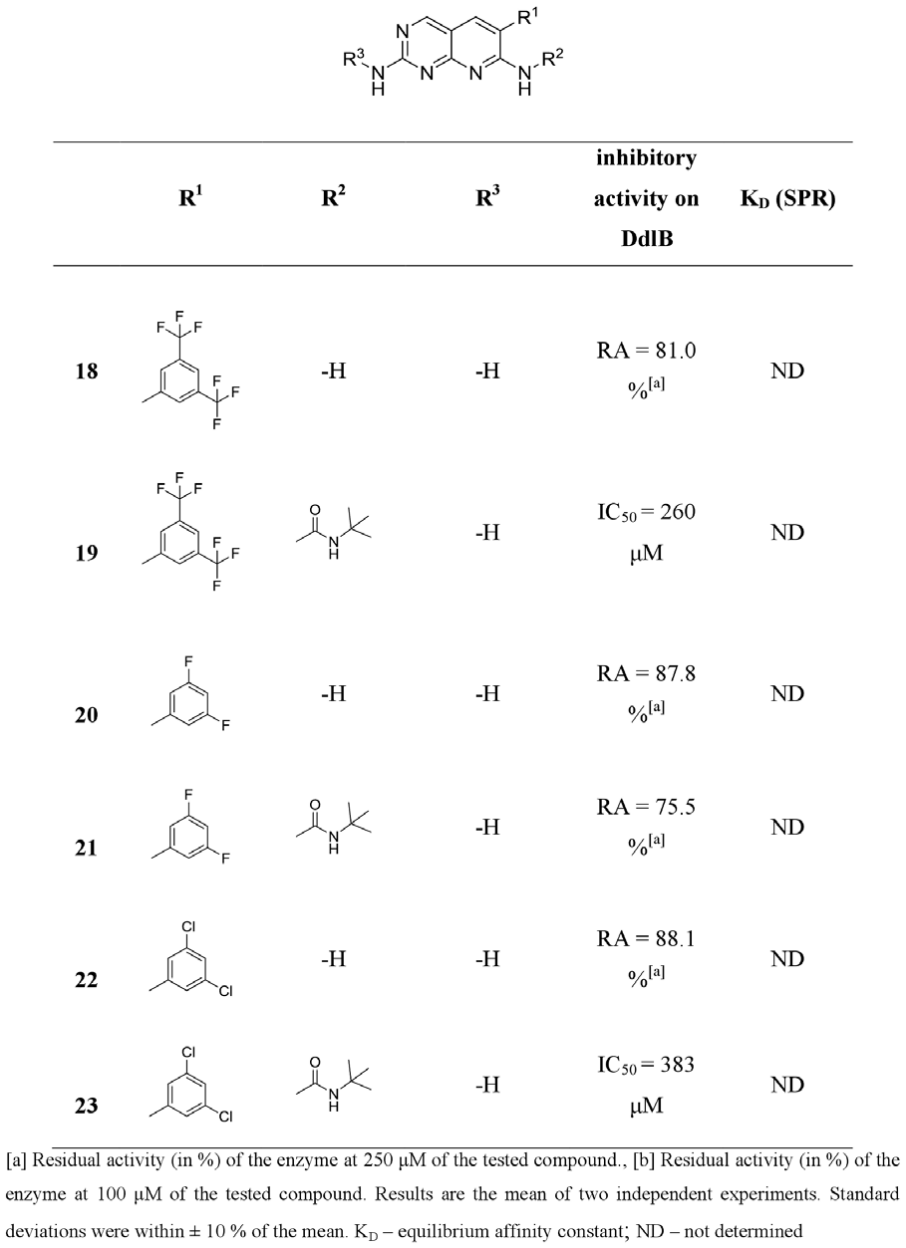
Inhibitory activities of pyridopyrimidines toward DdlB (continued).

**Figure 6:**
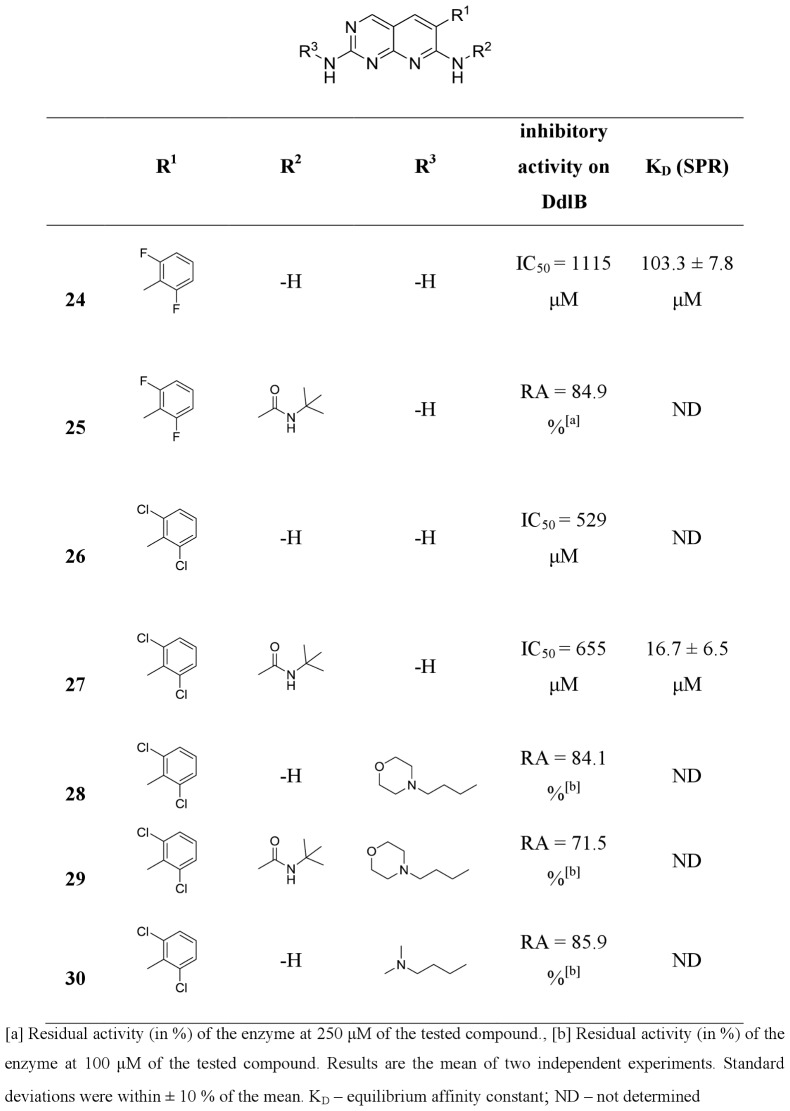
Inhibitory activities of pyridopyrimidines toward DdlB (continued).

**Figure 7:**
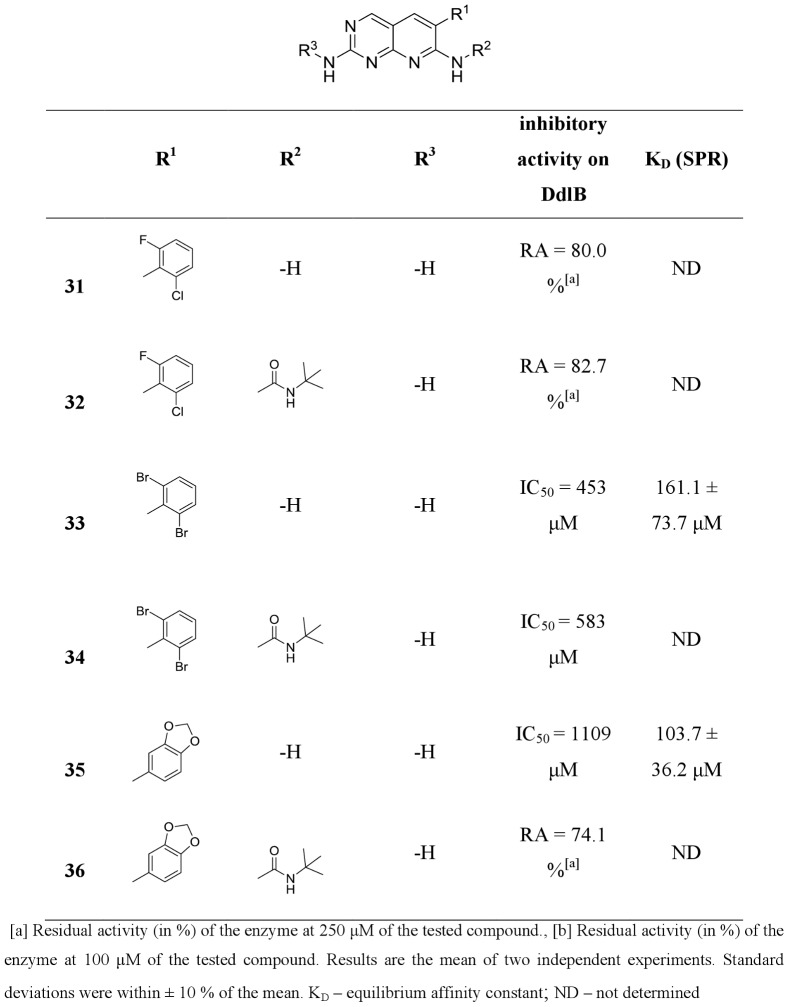
Inhibitory activities of pyridopyrimidines toward DdlB (continued).

We postulated that the pyridopyrimidine moiety could interact with the adenine-binding region in the ATP-binding site of our target enzyme and, since this core structure allows the introduction of different substituents onto three regions of the molecule, it offers a good opportunity to create additional interactions in the active site. In the first phase, we examined whether the selected scaffold shows any inhibition of DdlB. We prepared compound **33** and a series of additional nine 2,7-unsubstituted diamines (**11**, **13**, **18,** **20**, **22**, **24**, **26**, **31** and **35**) from commercially available or easily synthesized acetonitriles, and tested them for inhibition of the target enzyme (Figures 4, 5, 6, 7). Encouraged by the promising DdlB inhibitory activity of compound **33** and analogues, we assessed the role of N-2 and N-7 substitution on the inhibitory activity of target compounds. Corresponding N-7 *tert*-butylurea derivatives (**12**, **14**, **19**, **21**, **23**, **25**, **27, 32, 34** and **36**) were synthesized initially. The 3,5-dimethoxyphenyl- and 2,6-dichlorophenyl- [2,3-*d*]pyridopyrimidine derivatives were selected for further optimization due to the synthetic accessibility of corresponding acetonitriles and few compounds were designed and synthesized to explore the impact of N-2 substitution alone (**15**, **17**, **28** and **30**) and together with the reintroduction of *tert*-butyl urea on N-7 (**16** and **29**). Since the series of previously prepared compounds exhibited poor solubility, we introduced a basic aliphatic side chain at N-2 to improve aqueous solubility. Two aliphatic amines were selected, namely the 3-(dimethylamino)propylamine and the more bulky 4-(3-aminopropyl)morpholine, for exploration of the space around the distal amine.

### Chemistry

**Figure 8:**
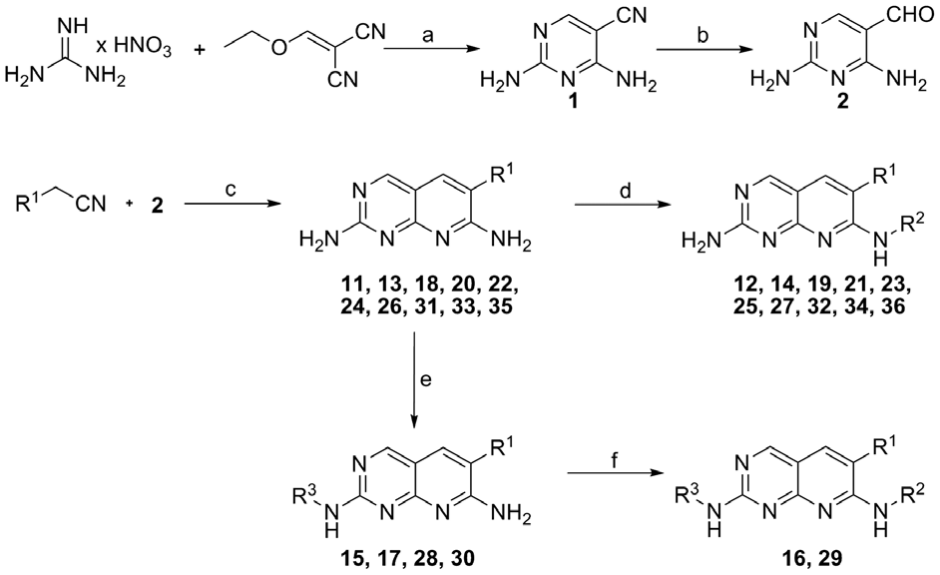
Reagents and conditions: (a) Na, EtOH, −5°C – room temp, 18 **h (b) Ra-Ni, 98–100% HCOOH (c) NaH, EtO(CH_2_)_2_OH, reflux, 4** **h.** (d) NaH, R_2_NCO/R_2_NCS, DMF, room temp, 18 h. (e) R^3^NH_2_, NH_2_SO_3_H, reflux, 42–72 h. (f) NaH, R_2_NCO/R_2_NCS, DMF, room temp, 18 h.

The syntheses of compounds reported in this paper are outlined in Figure 8 and described in details in Information S1. In the first step, 2,4-diamino-5-cyanopyrimidine (**1**) was prepared according to the method reported by Huber [30], by condensing guanidine nitrate with ethoxymethylenemalononitrile in ethanol in the presence of a significant amount of sodium ethoxide, to form the free guanidine base. The cyano group was reduced using Raney nickel as catalyst and 98% formic acid as solvent [31].

The resulting 2,4-diaminopyrimidine-5-carboxaldehyde (**2**) was condensed with commercially available or synthesized benzylnitriles (**7–10**) under basic conditions to give 2,7-diaminopyridopyrimidines (**11**, **13**, **18, 20**, **22**, **24**, **26**, **31, 33** and **35**) that were then treated further in two different ways. In the first stage, the diamine was acylated only at N-7, using 1 equivalent of basic NaH and 1 equivalent of isocyanate to give the final monosubstituted compound (**12**, **14**, **19**, **21**, **23**, **25**, **27, 32, 34** and **36**) with traces of bis-2,7-urea by-product [32]. Alternatively, the N-2 amine of the diamine was first substituted selectively in the presence of an acid such as sulfamic acid, with a refluxing nucleophilic aminoalkylamine as solvent (**15**, **17**, **28** and **30**). The latter was finally treated with 1 equivalent of NaH, followed by acylation with 1 equivalent of an acylating agent *tert*-butyl isocyanate to give the target urea compound (**16** and **29**).

**Figure 9:**
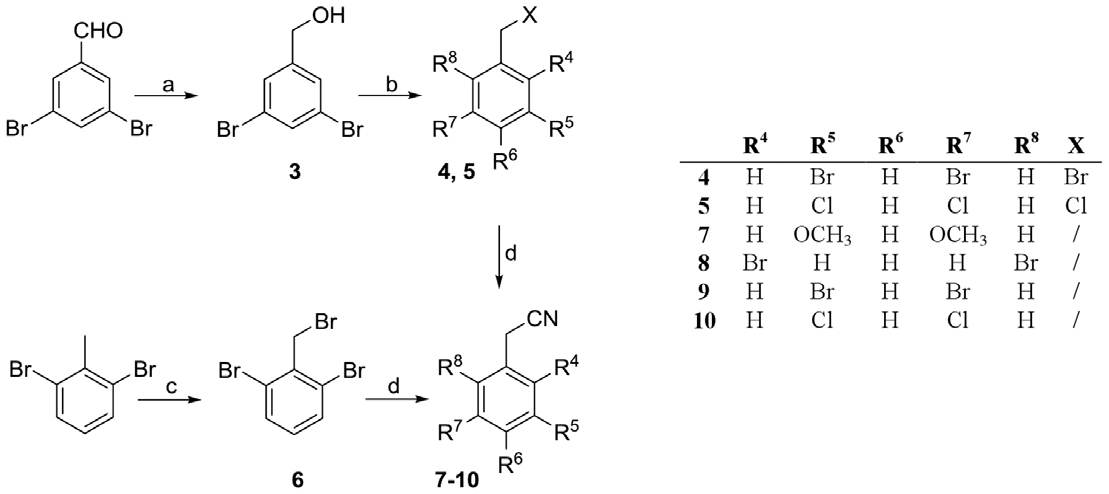
Reagents and conditions: (a) NaBH_4_, MeOH, room temp, 2 **h.** (b) CBr_4_, PPh_3_, THF, room temp, 16 h. (c) NBS, Bz_2_O_2_, CCl_4_, reflux, 4 h. (d) KCN, EtOH/H_2_O  = 4/1, reflux, 4 h.

The preparation of commercially unavailable phenylacetonitriles was achieved using two different approaches (Figure 9). In the first, the target compound was obtained from commercially available benzaldehydes in three steps. Reduction with sodium borohydride afforded the corresponding alcohol (**3**) [33] which was then converted to the respective bromide (**4**) by treatment with carbon tetrabromide and triphenylphosphine in THF [34], with subsequent cyanide displacement with KCN in a boiling mixture of ethanol and water. The initial step in the second approach was benzylic bromination of substituted toluene using *N*-bromosuccinimide in CCl_4_ to yield the bromide **6**, which was then converted to the corresponding cyanide **8** in the presence of KCN, as described previously [35].

### Biological activity

All of the synthesized 6-arylpyrido[2,3-*d*]pyrimidines **11–36** were assayed for *in vitro* inhibitory activity on *E. coli* DdlB by detecting orthophosphate generated during the enzymatic reaction using a Malachite green assay [36]. The results are presented as residual activities (RAs) of the enzyme in the presence of 100 or 250 μM of target compounds and as IC_50_ values for the most active compounds (Figures 4, 5, 6, 7). As a positive control in our assay we used D-cycloserine that exhibited IC_50_ value of 296 μM.

Three regions of the basic scaffold were targets for structure-activity relationship studies. Analogues with modifications of the 2-, 6-, and 7-positions were synthesized and evaluated for their inhibition of DdlB. The effects of phenyl substitution were investigated first. Compound **11** possessing an unsubstituted phenyl ring showed weak inhibition of the target enzyme, with an IC_50_ of 1140 µM. Disubstituting the meta positions of the phenyl ring did not lead to significant improvement of inhibitory potency (**13**, **18**, **20** and **22**), while, in contrary, disubstitution at the ortho positions resulted in the most potent inhibitor in the series of 6-arylpyrido[2,3-*d*]pyrimidine-2,7-diamines, with bromine introduced into the phenyl ring (**33**), which inhibited DdlB with an IC_50_ value of 453 μM. Incorporation of the fluorine in the phenyl ring did not have any impact on the inhibitory activity in the series of unsubstituted diamines (**20** and **24**) as well as in the next designed series (**21** and **25**) of 6-arylpyrido[2,3-*d*]pyrimidines, in which we examined the attributes of *tert*-butylurea substitution at the N-7 position. The inhibitory potency was significantly increased in the compounds with meta disubstitution at phenyl moiety in the 6-position. The 3′,5′-dimethoxy-substituted *tert*-butylurea derivative **14** with an IC_50_ value of 340 μM displays a 2.6-fold greater inhibitory potency as its corresponding diamine **13** and a similar trend can be seen for 3,5-bis(trifluoromethyl)- (compound **19**, IC_50_, 260 μM) and3′,5′-dichloro- *tert*-butylurea derivative **23** (IC_50_, 383 μM). On the other hand, if the *tert*-butylurea moiety was introduced at the N-7 position ofortho substituted 6-arylpyrido[2,3-*d*]pyrimidines, inhibition of the target enzyme was slightly reduced (**26** vs. **27**, **33** vs **34**).

The effect of N-2 substitution of the 2,7-diamino series of compounds on DdlB inhibition was investigated by incorporating basic aliphatic side chains at N-2 of the pyridopyrimidine scaffold. The introduction of a 3-(dimethylamino)propyl moiety reduced the DdlB inhibitory activity (**17** and **30**). An N-morpholinopropyl moiety, which was used for examination of steric constraints around the distal amine, had the same effect (**15** and **28**). However, reintroduction of *tert*-butylurea on the N-7 amine resulted in the most potent DdlB inhibitor in our studies, compound **16**, with an IC_50_ value of 133 μM.

Additionally we performed detailed inhibition studies for two selected compounds, namely the most potent inhibitor from 6-arylpyrido[2,3-*d*]pyrimidine-2,7-diamines, compound **33**, and compound **14**, which belongs to the series of *tert*-butylureas. Inhibition of DdlB was measured at different inhibitor and ATP concentrations in order to determine K_i_ values and the mode-of-action of both compounds. Kinetic analysis, as expected, revealed inhibition of the enzyme in an ATP-competitive mode for both compounds, **14** and **33**, with K_i_ values of 31±4 μM and 38±5 μM, respectively (Figure S1).

### Surface Plasmon resonance

**Figure 10:**
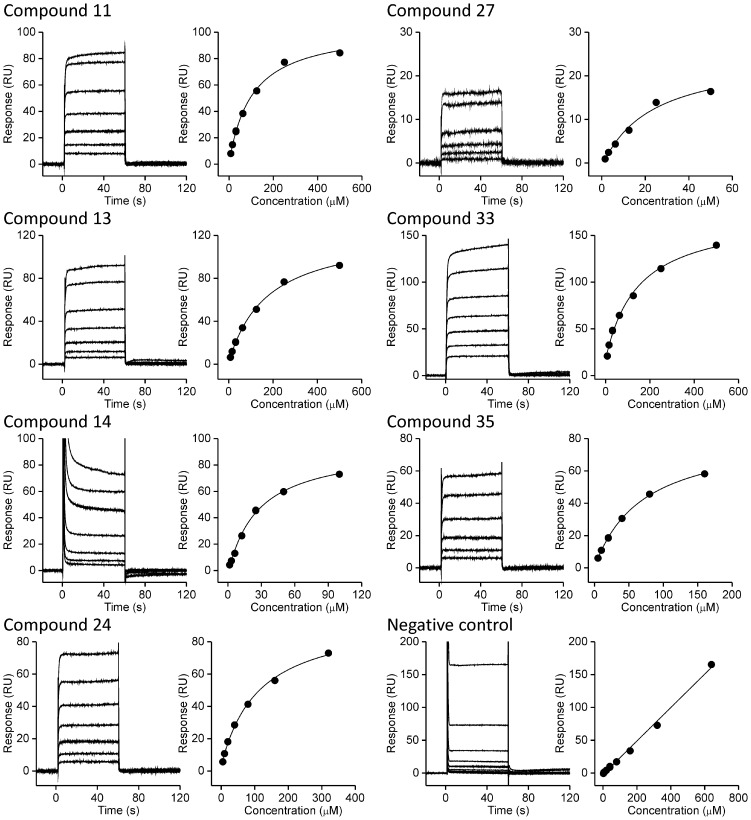
SPR analysis of compounds binding to DdlB. Different concentrations of compounds were tested for the binding (left panels in all cases). The binding curves (right panels) were generated by ploting steady-state response levels, i.e. at the end of the association phase, vs concentration of the injected compound. The KDs were obtained from fitting the data to the steady-state affinity model and are reported in Table 1. For each compound 3–5 independent titrations were performed.

The binding of the compounds **11**, **13**, **14**, **24**, **27**, **33,** and **35** to the target enzyme was confirmed using surface plasmon resonance (SPR) (Figure 10). Experiments were performed at conditions that enable long term stability of the enzyme. We found that in the buffer 50 mM Hepes, 150 mM NaCl, 5% DMSO, pH 8.0 the enzyme was stable for several days so that we could analyzed the binding of many compounds. The enzyme was covalently attached to the surface of a sensor chip CM5 and compounds were titrated over the surface. The binding of all the compounds was characterised by fast association and dissociation rates. Equilibrium affinity constants, estimated from the steady-state binding levels are in agreement with enzymatic studies and ITC. In addition, the binding of other compounds was tested but insufficient solubility at higher concentrations of analytes in the running buffer precluded analysis.

**Table 1:** Standard thermodynamic parameters (enthalpy, 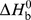, entropy, 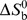, and Gibbs free energy, 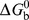) of the binding of **14** and **33** to DdlB at 37°C and the corresponding binding constants, *K*_b_, obtained by ITC.^[a]^

**compound**	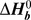 **/kcal mol^−1^**	***K*_b_/M^−1^**	***K*_d_/μM**	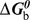 **/kcal mol^−1^**	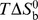 **/kcal mol^−1^**
**14**	−4	10^5^	10	−7	3
**33**	4	10^5^	10	−7	11

[a]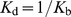, 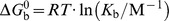, 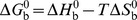.

### Isothermal titration calorimetry

Two of the compounds, **14** and **33**, were also tested by ITC to obtain thermodynamic parameters of the binding into DdlB active site. Heat changes upon binding of the ligand on the *E. coli* DdlB were measured directly, corrected for the heats of dilution, and the model function was fitted to the experimental data points (see the Experimental section).

**Figure 11:**
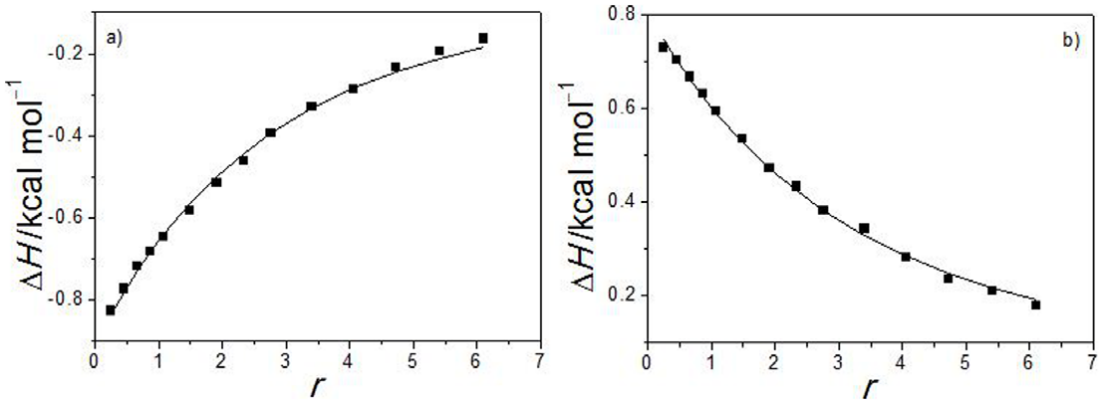
Calorimetric binding isotherms (▪) and the corresponding best fit model functions (–) for binding of a) 14 and b) 33 to DdlB at 37°C. r is the bound ligand to total protein concentration ratio.

The value of critical parameter for obtaining highly reliable thermodynamic data (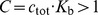) was not optimal due to low inhibitors solubility and quite low binding constants. Nevertheless, the conclusions below can be drawn. Experiments for the two compounds result in similar binding constants (*K*_b_≈10^5^ M^−1^) (Table 1), but the driving forces for the binding differ (Figure 11). The binding of compound **14** to DdlB results from favourable enthalpy and entropy contributions. In contrast, the binding of compound **33** is entropy driven, what is characteristic of a hydrophobic interaction (positive entropy change accompanying the release of ordered water molecules at the nonpolar surfaces in the bulk), and accompanied by positive enthalpy change. But still, compensation of the opposing effects at **33** results in similar 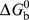 (and binding constant) as at **14**, although there are less specific interactions between **33** and the protein as at **14**. In other words, ITC experiments reveal comparable binding affinity of the tested compounds to DdlB active site; however more hydrogen bonds are formed upon binding of **14** obviously, what is also supported with the docking results.

### Docking

Plausible binding modes of the target compounds in the active site of *E. coli* DdlB were explored by docking the ligands using CDOCKER algorithm, as available in Accelrys Discovery Studio 3.0. The binding site was defined as a sphere with radius 13.9 Å around the centroid of the ADP molecule in the crystal structure of DdlB-ADP-phosphoryl phosphonate inhibitor ternary complex (PDB entry: 1IOV [13]). The defined binding site was large enough to include the ATP- and both D-Ala-binding sites of the DdlB active site. Prior to docking the potential DdlB inhibitors, CDOCKER docking protocol was validated by redocking ADP in the defined binding site. The program reproduced the experimentally determined binding mode of ADP with an all heavy atom RMSD of only 0.58 Å, which confirmed its suitability for prediction of binding modes of the designed DdlB ligands.

**Figure 12:**
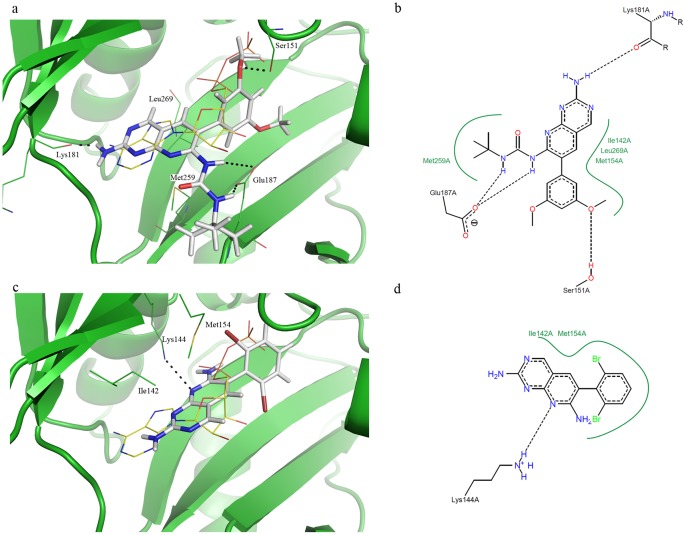
Superposition of ADP (PDB entry: 1IOV, in yellow lines) and docked pose of inhibitor a) 14 (in grey sticks) and c) 33 (in grey sticks) in the *E. coli* DdlB active site (in green). Schematic representation of interactions between b) 14 or d) 33 and *E. coli* DdlB active site residues as generated by PoseViewWeb [45].

Docking of inhibitors **14** and **33** in the *E. coli* DdlB active site (Figure 12) predicted their binding in the ATP-binding site, while both D-Ala-binding sites remained unoccupied. The pyrido[2,3-*d*]pyrimidine ring of **33** is predicted to bind in the adenine-binding pocket of the DdlB active site. Similar binding of the pyrido[2,3-*d*]pyrimidine ring has already been observed in the crystal structure of biotin carboxylase in complex with **33** (PDB entry 2V58).[29]. However, the binding mode of **33** in the biotin carboxylase active site differs from its predicted binding mode in the DdlB active site (Figure 12 and Figure S2). Compound **33** forms five hydrogen bonds with amino acid residues and additional hydrophobic interaction in the biotin carboxylase ATP-binding site (Figure S2), which results in the very potent inhibitory activity (IC_50_ less than 5 nM). In contrast, inhibitor **33** forms, according to the docking prediction, only one hydrogen bond with the side chain amino group of Lys144 and hydrophobic interactions with Ile142 and Met154 in the DdlB ATP-binding site, and inhibits the enzyme with an IC_50_ of 453 µM. Similarly, the pyrido[2,3-*d*]pyrimidine ring of **14** is seen to occupy the adenine-binding pocket of DdlB active site, but in an orientation different from that in the case of **33** (Figures 12a and 12c). A hydrogen bond is formed between the inhibitor amino group at position 2 and the carbonyl oxygen of Lys181. Additional hydrogen bonds are formed between the inhibitor urea NH groups and the Glu187 side chain carboxylate group, and between the inhibitor methoxy group and the Ser151 side chain hydroxyl group. Inhibitor **14** also forms additional hydrophobic contacts within the DdlB ATP-binding site. These docking results are in a good agreement with the results obtained by ITC, which showed mainly hydrophobic interaction as the driving force in binding **33** and favourable enthalpy and entropy contributions to the binding of **14**.

### Antibacterial activity

Selected DdlB inhibitors were tested for their antibacterial activity against two *E. coli* strains *E. coli* 1411 and *E. coli* AB734 (Table 2). Compounds **14**, **22**, **27** and **35** were inactive against all strains with MICs >256 μg/mL. Only 6-arylpyrido[2,3-*d*]pyrimidines with unsubstituted amino groups and disubstitution at the ortho position of the phenyl ring (compounds **24**, **26** and **33**) were found to inhibit both these strains, with MICs of 64 μg/mL and 32 μg/mL, respectively. Additionally, growth inhibition of efflux pump-deficient strains SM1411 (*acrAB* deficient derivative of 1411) [37] and ES100 (*tolC* deficient derivative of AB734) [38] was measured, and MICs were repeated for each strain in the presence of polymyxin B nonapeptide (PMBN) to assess the impact of outer-membrane permeability on antimicrobial activity [39]. Compounds **24**, **26** and **33** showed improvedMIC valuesagainst efflux-deficient, permeabilised cells, and compounds **16**, **19**, **23** and **34** (which are mono- or disubstituted 6-arylpyrido[2,3-*d*]pyrimidines) also showed weak antibacterial activity.

**Table 2:** Microbiological data for DdlB pyridopyrimidine-based inhibitors: MIC in μg/ml.

			SM1411	ES100	1411	AB734	SM1411	ES100
	1411	AB734	(*acrAB*)	(*tolC*)	+PMBN	+PMBN	+PMBN	+PMBN
**13**	>256	>256	>256	>256	>256	>256	128	64
**16**	>256	>256	>256	>256	>256	>256	128	128
**19**	>256	>256	>256	>256	>256	>256	>256	64
**23**	>256	>256	>256	>256	>256	>256	32	16
**24**	64	64	64	16	16	16	8	8
**26**	32	32	2	1	2	2	0,5	0,25
**33**	32	32	1	0,5	2	2	0,25	0,125
**34**	>256	>256	>256	4	32	32	4	0,5

The lack of correlation between enzyme inhibitor activity and antibacterial activity (Figures 4, 5, 6, 7 and Table 2) suggests that the antibacterial activities observed for the starting point compound **33** and the analogues **24** and **26** may primarily be due to inhibition of another bacterial target, and not DdlB.

### Conclusions

To conclude, we have designed, synthesized and evaluated a series of novel inhibitors of *E. coli* DdlB based on the 6-arylpyrido[2,3-*d*]pyrimidine scaffold. Best activity was achieved by disubstituted 6-arylpyrido[2,3-*d*]pyrimidine **16** (IC_50_ value of 133 μM) which demonstrated 3.4-fold improved IC_50_ value against DdlB compared to the lead compound **33**. For 7 selected compounds positive results from enzyme assays were additionally confirmed by surface plasmon resonance experiments, which show fast and tight binding of examined inhibitors to the DdlB. The mode-of-action was studied in detail for compounds **14** (IC_50_ value of 340 μM) and **33** (IC_50_ value of 453 μM) with a steady-state kinetic study, which revealed that both compounds, **14** and **33**, act as ATP competitive inhibitors of DdlB with K_i_ values of 31±4 μM and 38±5 μM, respectively. ITC data for the compounds **14** and **33** showed that the thermodynamic binding profile of compound **14** to DdlB elicited a favourable enthalpy and entropy contributions in comparison with the binding of compound **33**, which is entropy driven. Docking results are also in a good agreement with the results obtained by ITC. Some of the inhibitors exhibit antibacterial activity against *E.coli*, although their activity against wild-type strains is limited by outer membrane impermeability and efflux. It remains to be established whether the antibacterial effect results specifically from inhibition of DdlB in whole bacterial cells. The compounds in this study may serve as starting points for the development of bi-substrate inhibitors that incorporate both, an ATP-competitive and D-alanine competitive moieties, which would exhibit enhanced selectivity and potency profiles by preferentially inhibiting Ddl over kinases.

## Materials and Methods

### Chemistry

Chemicals were obtained from Acros, Aldrich Chemical Co., Apollo Scientific, and Fluka and used without further purification. Analytical thin-layer chromatography was performed on silica gel Merck 60 F_254_ precoated plates (0.25 mm), visualized with ultraviolet light, ninhydrin and 2,4-dinitrophenylhydrazine. Flash column chromatography was carried out on silica gel 60 (particle size 0.040–0.063 mm; Merck, Germany). Melting points were determined on a Reichert hot stage microscope and are uncorrected. ^1^H NMR spectra were recorded on a Bruker AvanceIII 400 MHz spectrometer at 295 K and 400 MHz, and are reported in ppm using solvent as internal standard (DMSO-d_6_ at 2.50 ppm, CDCl_3_ at 7.26 ppm). ^13^C NMR spectra were recorded on a Bruker Avance III 400 MHz spectrometer at 295 K and 100 MHz, and are reported in ppm using solvent as internal standard (DMSO-d_6_ at 39.5 ppm). Mass spectra data were recorded using a Q-Tof Premier (Waters-Micromass, Manchester, UK). HPLC analyses were performed on an Agilent Technologies HP 1100 instrument with a G1365B UV-vis detector, a G1316A thermostat, and a G1313A autosampler, using a Phenomenex Luna 5 μM C18 column (4.6 mm×150 mm) at flow rate of 1.0 mL/min. The eluent consisted of 0.1% trifluoroacetic acid in water (A) and methanol (B). Gradient was 20% B to 80% B in 20 min. The purity of the tested compounds was established to be ≥95%. All experimental procedures are described in Information S1.

### Protein expression and purification

The gene encoding DdlB from *E. coli* JM109 was amplified by PCR using the primers DdlBF5′ ACTGATAAAATCGCGGTCCTG 3′ and DdlBR 5′TTAGTCCGCCAGTTCCAGAATTCG′. The resulting product was cloned into pQE-30 UA (Qiagen), sequenced and transformed into *E. coli* BL21(λDE3). For overexpression, the resulting strain was grown and lysed as described in the pET manual (Novagen). The enzyme was expressed with an N-terminal polyhistidine tag and purified using a nickel affinity resin as described by the manufacturer (Novagen) [25].

### Colorimetric Inhibition Assay

The target compounds were tested for their ability to inhibit the D-Ala-adding activity of DdlB ligase with the colorimetric malachite green method in which orthophosphate generated during the reaction is measured. Each compound was tested in duplicate at 100, 250 or 500 μM in a mixture, final volume 50 μL, containing 50 mM Hepes (pH 8.0), 5 mM MgCl_2_, 10 mM (NH_4_)_2_SO_4_, 10 mM KCl, 700 μM D-Ala, 500 μM ATP, purified *His*-tagged DdlB (diluted in 50 mM Hepes (pH 7.2) and 1 mM DTT) and the test compound dissolved in DMSO. The final concentration of DMSO was 5% (v/v). The reaction mixture was incubated at 37°C for 20 min, then quenched with 100 μL of Biomol reagent and absorbance at 650 nm was measured after 5 min. To exclude possible promiscuous inhibitors, all compounds were tested in the presence of Triton-114 (0.005%). Residual activities were calculated relative to control assays without the compounds and with DMSO. IC_50_ values, the concentrations of the compounds at which the residual activities were 50%, were determined by measuring the residual activities in triplicate at seven different compound concentrations. K_i_ determinations were performed under similar conditions using D-Ala (10 mM), ATP (50, 100, 200, 300 and 500 μM) and inhibitor (50, 100, 200, 250, 350, 500 μM) with 20 min incubation at 37°C.

### Surface plasmon resonance (SPR)

The binding constants were obtained by using a Biacore T100 (Biacore, GE Healthcare, USA). The enzyme was immobilizated on the surface of the CM5 sensor chip in the running buffer without DMSO (50 mM Hepes, 150 mM NaCl, pH 7.4) according to the manufacturer's suggestions. The surface of the CM5 sensor chip was activated with a 7-min injection of the mixture N-hydroxysuccinimide and 1-ethyl-3-(3-dimethylpropyl)-carbodiimide as suggested by the manufacturer. Two 1 min injections of 50 μg/ml DdlB diluted in 10 mM Na-acetate, pH 4.0 were applied on the second flow cell. The first flow cell was left unmodified and served as a control for non-specific binding of tested compounds. The remaining active groups on the surface of both flow cells were deactivated with a 7-min injection of ethanolamine. The level of immobilized enzyme was around 10000 response units.

Each analyte was titrated, with 3 or 4 repetitions, using different concentrations according to their solubility in running buffer (50 mM Hepes, 150 mM NaCl, 5% DMSO, pH 7.4), usually from 0,781 μM to 500 μM. One concentration was repeated at the end of the assay in each dilution series, in order to check the surface stability and repeatability. The association lasted 60 seconds at a flow-rate 30 μl/min. No regeneration was needed. All experiments were performed at 25°C. Sensorgrams were double referenced by subtracting the signal obtained in the empty flow cell and the injection of the buffer. The steady-state response levels were fitted using the Steady State Affinity model in Biacore T100 Evaluation software.

### Isothermal titration calorimetry (ITC)

The heat changes upon binding were measured using a VP-ITC microcalorimeter from MicroCal, LLC (Northampton, MA, USA). Purified DdlB stock solution in 20% glycerol was dialysed for 40 hours at 4°C (50 mM Hepes, pH = 8.0, 10 mM KCl, 10 mM (NH_4_)_2_SO_4_, 5 mM MgCl_2_, and 0.01% Triton X-114) prior to titration. The ligand solutions were prepared by adding a stock solution of **14** or **33** in DMSO to the dialysis buffer to obtain final 50 μM solution of the DdlB inhibitor in 5% DMSO. Successive aliquots (11–31 μl) of the degassed ligand solution were injected at 5–8 min intervals by a motor driven syringe (300 μl) into the degassed DdlB solution (approximately 2 μM, 5% DMSO) in the calorimeter cell (V = 1.386 ml) with constant stirring (300 rpm) at 37°C. At the end of experiment the final ratio of ligand:enzyme in the titration cell was about 6∶1. The titration data was further corrected for the heat changes observed in the control titration of ligand solution into the dialysis buffer, 5% DMSO, alone. The experimental data was analysed using the Origin 7.0 software provided by MicroCal and fitted by a home written program, based on the non-linear Levenberg–Marquardt χ^2^ regression procedure, assuming a single binding site model (two parameter fit) [40]:
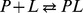


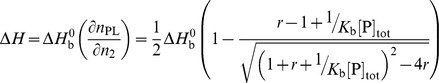
where P, L, and PL represent DdlB, ligand, and DdlB-ligand complex, Δ*H* and 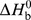 are the measured enthalpy upon aliquot addition and the standard enthalpy change of the binding reaction, 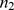 and 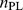 represent the total number of moles of ligand and DdlB-ligand complex in the titration cell, *r* is the bound ligand to total protein concentration,[P]_tot_, ratio and *K*_b_ is the binding constant. A detailed explanation of the applied model equation is given in the Information S2.

### Molecular docking

#### Ligand and protein preparation

Three-dimensional models of the target compounds were built from a standard fragment library, and their geometries optimized using the CHARMm force field [41] with MMFF94 [42] partial atomic charges. The Smart Minimizer algorithm, as available in Accelrys Discovery Studio 3.0 (DS) [43] running on a workstation with Intel Core i7 860 CPU processor, 8 GB RAM, two 750 GB hard drives and an Nvidia GT220 GPU graphic card, running Centos 5.5, was used for energy minimization until the gradient value was smaller than 0.001 kcal mol^−1^ Å^−1^. Molecular docking calculations were performed using Dock Ligands (CDOCKER) protocol available in DS. Prior to docking, target enzyme DdlB, co-crystallized with ADP and phosphoryl phosphonate-based inhibitor (PDB code: 1IOV [13]), was prepared using Prepare Protein protocol in DS, during which the protein was typed with CHARMm force field, hydrogen atoms were added, water molecules were deleted and protonation states were assigned. Binding site was defined as a sphere with radius 13.9 Å around the centroid of the ADP molecule (x = 19.504, y = 3.704, z = 36.555), which was large enough to accommodate the ATP and both D-Ala binding sites.

#### Docking validation and ligand docking

In order to validate CDOCKER as a suitable docking program for the binding mode prediction of the designed potential DdlB inhibitors, ADP was docked in the defined DdlB active site. A set of 10 starting random conformations was generated from the initial ligand structure through 1000 steps (1 fs/step) of high temperature MD with target temperature set to 1000 K. Electrostatic interactions were included in the random structure generation. For each of the MD-generated ligand conformations, 10 rigid-body rotations about its centre of mass were used as the initial conformations in the vicinity of the binding site. MD simulated annealing (SA) was then used, starting from these initial binding site conformations, to search for low energy conformations of the ligand in the defined binding site. The heating phase of SA consisted of 2000 steps (1 fs/step) of heating from temperature 300 K to 700 K, followed by 5000 steps (1 fs/step) of cooling phase to cool back from 700 K to 300 K. A final minimization of the ligand in the rigid receptor, using full potential energy terms, was performed in CHARMm force field. For each final pose, the CHARMm energy and the interaction energy alone were calculated. The poses were sorted by CHARMm energy and the 100 best top scoring poses were retained. The ten best docking poses were inspected visually and compared to the binding mode of ADP in the crystal structure of the DdlB-ADP complex (PDB code: 1IOV). The best ranked CDOCKER-calculated ADP conformation in the DdlB active site had an all heavy atom RMSD value of only 0.58 Å compared to the experimentally determined conformation of ADP in the DdlB active site.

Target compounds were docked to the DdlB active site using Dock Ligands (CDOCKER) protocol, with the same parameters as for the validation of the docking protocol with the ADP.

### Microbiological evaluation

Minimum inhibitory concentrations (MICs) were determined by broth microdilution against *E. coli* 1411, SM1411 (*acrAB* deficient derivative of 1411) [37], AB734 and ES100 (*tolC* deficient derivative of AB734) [38] according to CLSI guidelines [44]. MICs were repeated for each strain in the presence of polymyxin B nonapeptide (4 μg/ml) to assess the impact of outer-membrane permeability on antimicrobial activity [39].

## Supporting Information

Figure S1 **Kinetic analysis of DdlB inhibition of 14 (a); Kinetic analysis of DdlB inhibition of 33 (b).** Data were fitted for competitive, noncompetitive and uncompetitive inhibition models using SigmaPlot 11.0 software and K_i_ values for the best fitted model were calculated.
(TIF)

Figure S2 **Superposition of** ***E. coli*** **biotin carboxylase (PDB entry: 2V58, in yellow) and** ***E. coli*** **DdlB (PDB entry: 1IOV, in green) crystal structures.** Inhibitor 33 from the biotin carboxylase crystal structure is presented in yellow sticks, while its CDOCKER-calculated binding mode in the DdlB active site is shown in grey sticks (a); Schematic representation of interactions between **33** and biotin carboxylase active site residues as generated by PoseViewWeb [45] (b).
(TIF)

Table S1 **Structurally similar ATP-binding sites to Ddl ATP-binding site as obtained by ProBiS** [**46**] **.**
(DOCX)

Information S1 **Chemistry-experimental procedures.**
(DOCX)

Information S2 **Isothermal titration calorimetry.**
(DOCX)
