# Supplementary figures and images for: 6-Arylpyrido[2,3-d]pyrimidines as Novel ATP-Competitive Inhibitors of Bacterial D-Alanine:D-Alanine Ligase
Source: PLoS One. 2012 Aug 2;7(8):e39922. doi: 10.1371/journal.pone.0039922 (PMC3410885; doi:10.1371/journal.pone.0039922)

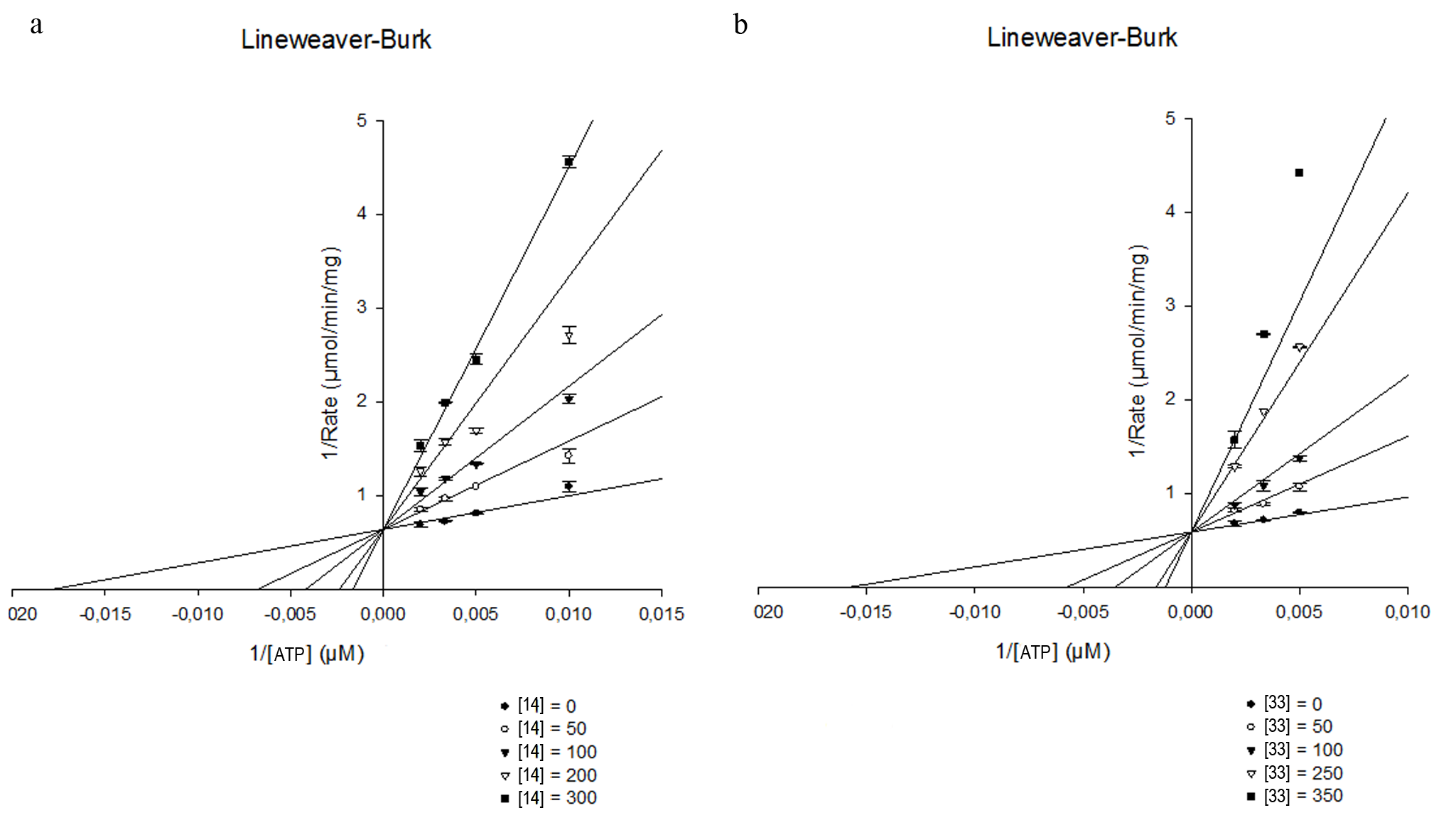

Supplement: Figure S1 — Kinetic analysis of DdlB inhibition of 14 (a); Kinetic analysis of DdlB inhibition of 33 (b). Data were fitted for competitive, noncompetitive and uncompetitive inhibition models using SigmaPlot 11.0 software and Ki values for the best fitted model were calculated. (TIF) [file pone.0039922.s001.tif]

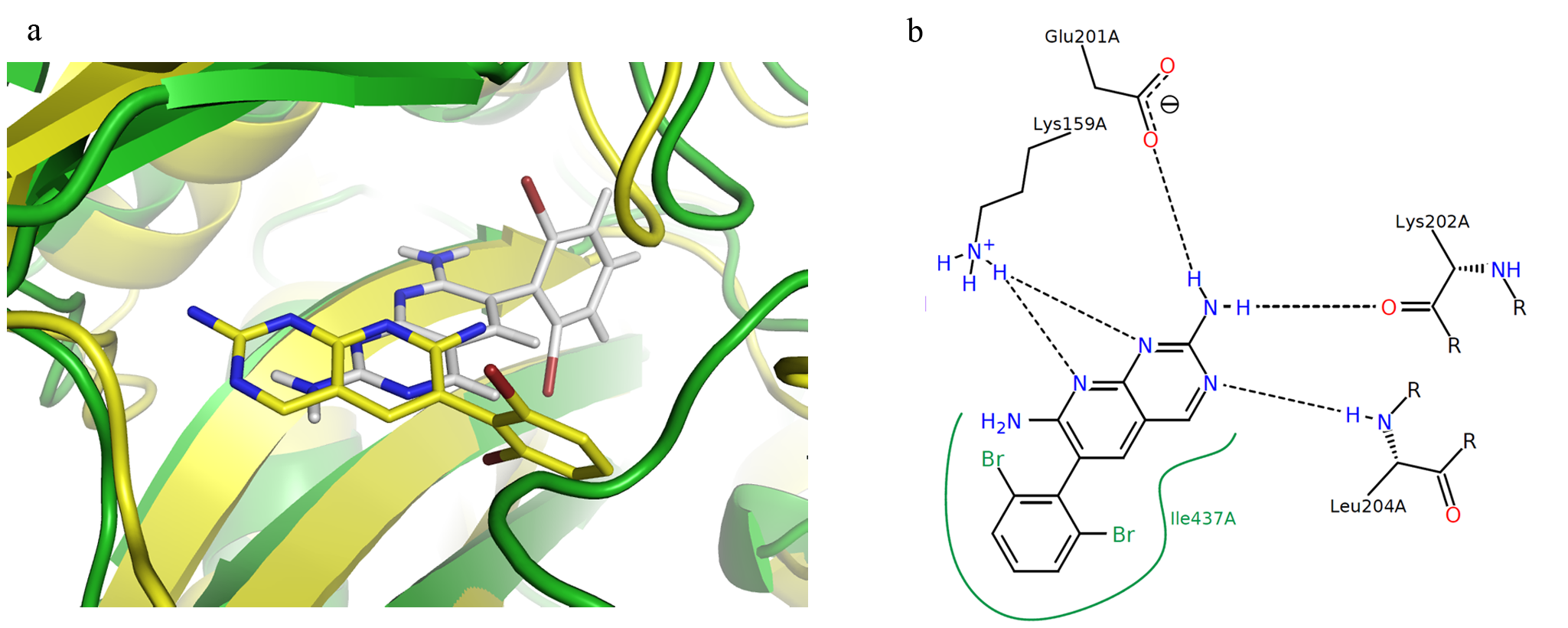

Supplement: Figure S2 — Superposition of E. coli biotin carboxylase (PDB entry: 2V58, in yellow) and E. coli DdlB (PDB entry: 1IOV, in green) crystal structures. Inhibitor 33 from the biotin carboxylase crystal structure is presented in yellow sticks, while its CDOCKER-calculated binding mode in the DdlB active site is shown in grey sticks (a); Schematic representation of interactions between 33 and biotin carboxylase active site residues as generated by PoseViewWeb [45] (b). (TIF) [file pone.0039922.s002.tif]
