# Supplementary material for: 6-Arylpyrido[2,3-d]pyrimidines as Novel ATP-Competitive Inhibitors of Bacterial D-Alanine:D-Alanine Ligase
Source: PLoS One. 2012 Aug 2;7(8):e39922. doi: 10.1371/journal.pone.0039922 (PMC3410885; doi:10.1371/journal.pone.0039922)
Supplement: Table S1 — Structurally similar ATP-binding sites to Ddl ATP-binding site as obtained by ProBiS [46] . (DOCX) [file pone.0039922.s003.docx]

| **PDB ID code** | **Number of aligned residues** | ***E*-value^a^** | **Enzyme^b^** | **Classification** | **Source** | |
| --- | --- | --- | --- | --- | --- | --- |
| **1iow** | 300 |  | D-alanine-D-alanine ligase – **query enzyme** | ligase | *Escherichia coli* | |
| 3i12 | 117 | 8.73e-71 | D-alanine-D-alanine ligase A | ligase | *Salmonella typhimurium* | |
| 2i87 | 117 | 3.75e-69 | D-alanine-D-alanine ligase | ligase | *Staphylococcus aureus* | |
| 3e5n | 112 | 4.21e-47 | D-alanine-D-alanine ligase A | ligase | *Xanthomonas oryzae* | |
| 2zdh | 96 | 9.97e-44 | D-alanine-D-alanine ligase | ligase | *Thermus thermophilus* | |
| 3k3p | 89 | 6.25e-53 | D-alanine-D-alanine ligase | ligase | *Streptococcus mutans* | |
| 1e4e | 80 | 1.42e-54 | D-alanyl-D-lactate ligase (VanA) | ligase | *Enterococcus faecium* | |
| 3lwb | 76 | 2.34e-60 | D-alanine-D-alanine ligase | ligase | *Mycobacterium tuberculosis* | |
| 1ehi | 68 | 2.45e-56 | D-alanyl-D-lactate ligase (VanA) | ligase | *Leuconostoc mesenteroides* | |
| 1a9x | 54 | 6.74e-15 | carbamoyl phosphate synthetase | transferase | *Escherichia coli* | |
| 2w70 | 52 | 2.19e-21 | biotin carboxylase | ligase | *Escherichia coli* | |
| 2x8u | 44 | 1.54e-06 | serine palmitoyltransferase | transferase | *Sphingomonas wittichii* | |
| 3kal | 40 | 6.23e-11 | homoglutathione synthetase | ligase | *Glycine max* | |
| 2pvp | 35 | 1.86e-30 | D-alanine-D-alanine ligase | ligase | *Helicobacter pylori* | |
| 2vpq | 35 | 4.59e-17 | acetyl-CoA carboxylase | ligase | *Staphylococcus aureus* | |
| 3eth | 32 | 5.37e-20 | phosphoribosylaminoimidazole carboxylase ATPase subunit | lyase | *Escherichia coli* | |
| 2vqd | 32 | 2.42e-13 | biotin carboxylase | ligase | *Pseudomonas aeruginosa* | |
| 2yw2 | 30 | 1.10e-10 | phosphoribosylamine--glycine ligase | ligase | *Aquifex aeolicus* | |
| 2dwc | 30 | 1.89e-08 | hypothetical phosphoribosylglycinamide formyl transferase | transferase | *Pyrococcus horikoshii ot3* | |
| 1ulz | 29 | 3.29e-10 | pyruvate carboxylase | ligase | *Aquifex aeolicus* | |
| 2dzd | 29 | 2.62e-09 | pyruvate carboxylase | ligase | *Geobacillus thermodenitrificans* | |
| 1vkz | 27 | 3.87e-14 | phosphoribosylamine--glycine ligase | ligase | *Thermotoga maritima* | |
| 3df7 | 27 | 1.12e-17 | putative ATP-grasp superfamily protein | structural genomics | *Archaeoglobus fulgidus* | |
| 2yrx | 27 | 1.05e-07 | phosphoribosylglycinamide synthetase | ligase | *Geobacillus kaustophilus* | |
| 2qk4 | 25 | 9.67e-16 | **glycinamide ribonucleotide synthase** | ligase | *Homo sapiens* | |
| 3glk | 25 | 7.02e-14 | **acetyl-CoA carboxylase** | ligase | *Homo sapiens* | |
| 3k5i | 25 | 6.70e-08 | phosphoribosyl-aminoimidazole carboxylase | lyase | *Aspergillus clavatus* | |
| 1kjq | 24 | 3.14e-09 | phosphoribosylglycinamide formyltransferase 2 | transferase | *Escherichia coli* | |
| 3fdd | 24 | 6.28e-08 | L-aspartate-beta-decarboxylase | lyase | *Pseudomonas dacunhae* | |
| 1m0w | 23 | 4.10e-12 | glutathione synthetase | ligase | *Saccharomyces cerevisiae* | |
| 2o8m | 23 | 3.09e-05 | protease | viral protein | *Hepatitis c virus* | |
| 1xg2 | 21 | 1.90e-05 | pectinesterase 1 | hydrolase | *Solanum lycopersicum* | |
| 2cfg | 21 | 5.24e-05 | amine oxidase | oxidoreductase | *Arthrobacter globiformis* | |
| 3mjf | 20 | 3.99e-11 | phosphoribosylamine--glycine ligase | ligase | *Yersinia pestis* | |
| 1gee | 20 | 3.97e-06 | glucose dehydrogenase | oxidoreductase | *Bacillus megaterium* | |
| 2hgs | 19 | 1.06e-08 | glutathione synthetase | ligase | *Homo sapiens* | |
| 2fhf | 19 | 3.34e-08 | pullulanase | hydrolase | *Klebsiella aerogenes* | |
| 3hbl | 18 | 1.43e-07 | pyruvate carboxylase | ligase | *Staphylococcus aureus* | |
| 2pn1 | 18 | 5.54e-12 | carbamoylphosphate synthase | ligase | *Exiguobacterium sibiricum* | |
| 3lul | 18 | 4.08e-06 | 4-amino-4-deoxychorismate lyase | lyase | *Legionella pneumophila* | |
| 1uc8 | 17 | 1.85e-11 | lysine biosynthesis enzyme | biosynthetic protein | *Thermus thermophilus* | |
| 3fi8 | 17 | 1.91e-06 | choline kinase | transferase | *Plasmodium falciparum* | |
| 1h72 | 16 | 1.86e-08 | homoserine kinase | transferase | *Methanococcus jannaschii* | |
| 1vpk | 16 | 3.11e-05 | DNA polymerase III, beta subunit | transferase | *Thermotoga maritima* | |
| 3n6r | 15 | 1.83e-06 | propionyl-CoA carboxylase | ligase | *Ruegeria pomeroyi* | |
| 3btp | 15 | 6.48e-09 | single-strand DNA-binding protein | DNA binding protein | *Agrobacterium tumefaciens* | |
| 3e5d | 15 | 4.83e-07 | putative glyoxalase I | structural genomics | *Listeria monocytogenes* | |
| 3mtk | 15 | 1.64e-07 | diguanylate cyclase/phosphodiesterase | transferase | *Caldicellulosiruptor saccharolyticus* | |
| 2vqe | 14 | 9.29e-09 | 30S ribosomal protein S4 | ribosome | *Thermus thermophilus* | |
| 1sjw | 14 | 5.48e-07 | nogalonic acid methyl ester cyclase | lyase | *Streptomyces nogalater* | |
| 3c5i | 14 | 2.49e-07 | choline kinase | transferase | *Plasmodium knowlesi* | |
| 2d16 | 14 | 7.61e-05 | hypothetical protein PH1918 | structural genomics | *Pyrococcus horikoshii* | |
| 3mwd | 13 | 2.95e-07 | **ATP-citrate synthase** | transferase | *Homo sapiens* | |
| 2z04 | 13 | 1.07e-05 | phosphoribosylaminoimidazole carboxylase ATPase subunit | lyase | *Aquifex aeolicus* | |
| 1wr2 | 13 | 7.91e-05 | hypothetical protein PH1789 | structural genomics | *Pyrococcus horikoshii* | |
| 1lsh | 12 | 1.04e-05 | lipovitellin | lipid binding protein | *Ichthyomyzon unicuspis* | |
| 3g8y | 12 | 3.17e-06 | SusD/RagB-associated esterase-like protein | hydrolase | *Bacteroides vulgatus* | |
| 3c9h | 12 | 5.16e-06 | ABC transporter, substrate binding protein | transport protein | *Agrobacterium tumefaciens* | |
| 3nng | 12 | 6.43e-06 | uncharacterized protein | structural genomics | *Bacteroides fragilis* | |
| 2ns1 | 12 | 6.78e-06 | nitrogen regulatory protein P-II 2 | signaling protein | *Escherichia coli* | |
| 1u04 | 12 | 1.17e-05 | hypothetical protein PF0537 | hydrolase | *Pyrococcus furiosus* | |
| 1uyn | 12 | 3.23e-06 | autotransporter | membrane protein | *Neisseria meningitidis* | |
| 1dpg | 11 | 7.38e-06 | glucose 6-phosphate dehydrogenase | oxidoreductase | *Leuconostoc mesenteroides* | |
| 3a4u | 11 | 7.61e-05 | **protein ERGIC-53** | protein transport | *Homo sapiens* | |
| 2crp | 11 | 2.28e-06 | **regulator of G-protein signaling 5** | signaling protein | *Homo sapiens* | |
| 1czd | 11 | 3.83e-05 | DNA polymerase accesory protein G45 | gene regulation | *Enterobacteria phage t4* | |
| 3c2u | 11 | 4.55e-05 | xylosidase/arabinosidase | hydrolase | *Selenomonas ruminantium* | |
| 3cpx | 11 | 7.29e-05 | aminopeptidase, M42 family | hydrolase | *Cytophaga hutchinsonii* | |
| 3kfu | 11 | 4.72e-05 | aspartyl/glutamyl-tRNA(Asn/Gln) amidotransferase subunit B | ligase | *Thermus thermophilus* | |
| 2o90 | 10 | 3.84e-05 | dihydroneopterin aldolase | lyase | *Escherichia coli* | |
| 3bk6 | 10 | 7.52e-05 | PH stomatin | membrane protein | *Pyrococcus horikoshii* | |
| 3e6u | 10 | 7.95e-07 | **LanC-like protein 1** | signaling protein | *Homo sapiens* | |
| 1osm | 10 | 5.71e-05 | osmoporin OmpK36 | membrane protein | *Klebsiella pneumoniae* | |
| 1sgv | 9 | 2.48e-05 | tRNA pseudouridine synthase B | lyase | *Mycobacterium tuberculosis* | |
| 1e5k | 9 | 7.22e-07 | molybdopterin-guanine dinucleotide biosynthesis protein A | transferase | *Escherichia coli* | |
| 3cnr | 9 | 9.06e-05 | type IV fimbriae assembly protein | unknown function | *Xanthomonas axonopodis* | |
| 3cit | 9 | 3.65e-05 | sensor histidine kinase | transferase | *Pseudomonas syringae* | |
| ^a^*E*-value – expectation value; ^b^Human enzymes are written in bold. | | | | | |  |
