# Supplementary material for: 6-Arylpyrido[2,3-d]pyrimidines as Novel ATP-Competitive Inhibitors of Bacterial D-Alanine:D-Alanine Ligase
Source: PLoS One. 2012 Aug 2;7(8):e39922. doi: 10.1371/journal.pone.0039922 (PMC3410885; doi:10.1371/journal.pone.0039922)
Supplement: Information S1 — Chemistry-experimental procedures. (DOCX) [file pone.0039922.s004.docx]

**Chemistry - experimental procedures:**

**Synthesis of 2,4-diaminopyrimidine-5-carbonitrile (1)**

Guanidine nitrate (12.2 g, 0.099 mol) was dissolved in a freshly prepared, cold solution (5 °C) of sodium ethoxide (2.3 g, 0.100 mol) in 70 ml absolute ethanol. To complete the formation of free guanidine base, the reaction mixture was kept stirred for an additional 15 minutes. The precipitated sodium nitrate was filtered, washed with 15 ml of absolute ethanol and the colourless filtrate with combined washings cooled to -5 °C. Ethoxymethylene malononitrile (12.0 g, 0.118 mol) was added in portions for 15 min, keeping the reaction mixture below 15 °C by cooling. Immediate reaction took place, turning the solution yellow. The temperature was then allowed to rise and the reaction mixture was stirred overnight at room temperature. After removal of about half the ethanol the solid precipitate was filtered, washed with cold ethanol, dissolved in glacial acetic acid, and treated with charcoal. After filtration, 25% ammonia solution was added until the filtrate became neutral. The product was filtered, washed with cold water and dried to yield 8.2 g (yield: 61%) of 2,4-diaminopyrimidine-5-carbonitrile, **1,** as a yellow solid; mp 313-315 °C (lit[47] 318 °C). ^1^H NMR (400 MHz, DMSO-d_6_): δ 6.92 (br s, 2H, NCNH_2_N), 7.10 (br s, 2H, NCNH_2_C), 8.16 (s, 1H, Ar-H) ppm. HRMS (ESI): m/z [M + H]^+^ calcd for C_5_H_5_N_5_, 136.1325; found, 136.1329.

**Synthesis of 2,4-diaminopyrimidine-5-carbaldehyde (2)**

To a solution of **1** (4.0 g, 0.030 mol) in 50 ml 98% formic acid, Raney nickel (4.5 g of 50% slurry with water) was added and the reaction mixture refluxed overnight at 120 °C. After the reaction was complete, it was filtered and washed with 10 ml formic acid. The filtrate and washings were collected together and concentrated under reduced pressure. The resulting residue was suspended in 25% ammonia solution, stirred with cooling for 15 min to give a free flowing solid product, which was filtered and washed with water. After recrystallization from water and drying, 2,4-diaminopyrimidine-5-carbaldehyde **2** was obtained as a pale yellow solid (3.2 g, yield: 78%); mp 273-275 °C (lit[48] 271 °C). ^1^H NMR (400 MHz, DMSO-d_6_): δ 7.13 (br d, 2H, NCNH_2_N), 7.69 (br d, 2H, NCNH_2_C), 8.31 (s, 1H, Ar-H), 9.45 (s, 1H, CHO) ppm. HRMS (ESI): m/z [M + H]^+^ calcd for C_5_H_6_N_4_O, 139.1295; found, 139.1301.

**Synthesis of (3,5-dibromophenyl)methanol (3)**

3,5-Dibromobenzaldehyde (1.0 g, 3.79 mmol) was dissolved in methanol (50 ml) and the solution cooled to -10 °C. Sodium borohydride (0.288 g, 7.58 mmol) was added in portions and the reaction mixture stirred for 2 h. The reaction was quenched by the addition of saturated aqueous NH_4_Cl, the volatiles were removed *in vacuo* and the residue was extracted with EtOAc. The organic layer was washed with brine, dried over Na_2_SO_4_ and the solvent was removed under reduced pressure to give the pure product as a white solid (yield: 94 %); mp 130-132 °C (lit[49] 135-136 °C). ^1^H NMR (400 MHz, DMSO-d_6_): δ 4.78 (s, 2H, CH_2_), 7.53 (d, *J* = 1.8 Hz , 2H, Ar-H-2,6), 7.69 (t, *J* = 1.8 Hz ,1H, Ar-H-4) ppm. HRMS (ESI): m/z [M + H]^+^ calcd for C_7_H_6_Br_2_O, 266.8875; found, 266.8871.

**Synthesis of 1,3-dibromo-5-(bromomethyl)benzene (4)**

A solution of triphenylphosphine (1.2 g, 4.69 mmol) in dry THF (10 ml) was added dropwise to a solution of **3** (1.0 g, 3.76 mmol) and carbon tetrabromide (1.6 g, 4.69 mmol) in dry THF (10 ml). The reaction mixture was stirred at room temperature overnight. The solvent was removed under reduced pressure and the residue purified by flash column chromatography using ethylacetate/hexane (1/10) as eluent to yield the pure product as a white solid (yield: 88%); mp 99-101 °C (lit.[50] 95-96 °C). ^1^H NMR (400 MHz, DMSO-d_6_): δ 4.69 (s, 2H, CH_2_), 7.72 (d, *J* = 1.8 Hz, 2H, Ar-H-2,6), 7.81 (t, *J* = 1.8 Hz ,1H, Ar-H-4) ppm. HRMS (ESI): m/z [M + H]^+^ calcd for C_7_H_5_Br_3_, 329.8347; found, 329.8342.

**Synthesis of 1,3-dichloro-5-(chloromethyl)benzene (5)**

(3,5-dichlorophenyl)methanol (1.5 g, 8.47 mmol) was dissolved in THF (50 ml) and SOCl_2_ (7 ml) was added. After the reaction was complete, ice cold water was added to the reaction mixture and the solution was extracted with EtOAc (3 × 30 ml). The organic layer was dried over Na_2_SO_4_ and the solvent removed under reduced pressure to obtain the pure product as a white solid (yield: 84 %). mp 39-40 °C (lit[51] 36 °C). ^1^H NMR (400 MHz, DMSO-d_6_): δ 3.59 (s, 2H, CH_2_), 7.46 (d, *J* = 1.9 Hz, 2H, Ar-H-2,6), 7.59 (t, *J* = 1.9 Hz ,1H, Ar-H-4) ppm. HRMS (ESI): m/z [M + H]^+^ calcd for C_7_H_5_Cl_3_, 196.4697; found, 196.4705.

**Synthesis of 1,3-dibromo-2-(bromomethyl)benzene (6)**

N-bromosuccinimide (3.2 g, 18.2 mmol) and dibenzoyl peroxide (0.3 g, 1.24 mmol) were added to a solution of 1,3-dibromo-2-methylbenzene (4.1 g, 16.5 mmol) in CCl_4_ and the reaction mixture refluxed for 4 h. After the reaction was complete, it was filtered and solvent was removed under reduced pressure to yield the pure product as a white solid (yield: 86 %). mp 86 °C (lit[52] 80-81 °C). ^1^H NMR (400 MHz, DMSO-d_6_): δ 4.56 (s, 2H, CH_2_), 7.53 (t, *J* = 8.0 Hz, 1H, Ar-H-4), 7.99 (d, *J* = 8.0 Hz, 2H, Ar-H-3,5) ppm. HRMS (ESI): m/z [M + H]^+^ calcd for C_7_H_5_Br_3_, 329.8359; found, 329.8360.

**Synthesis of Compounds 7, 8, 9 and 10**

To a solution of **4**, **5,** **6** or 1-(chloromethyl)-3,5-dimethoxybenzene (3.0 mmol) in ethanol/ water (4/1) KCN (0.589 g, 9.0 mmol) was added and the reaction mixture refluxed for 4 h. It was then poured into water and extracted with Et_2_O (3 × 30 ml). The organic layer was dried over Na_2_SO_4_ and the solvent removed under reduced pressure, giving a residue that was purified with flash column chromatography using ethylacetate/hexane (1/3) as eluent.

*2-(3,5-dimethoxyphenyl)acetonitrile (7)*

Yield: 87 %; off-white crystals; mp 83-86 °C (lit[53] 87-88 °C). ^1^H NMR (400 MHz, DMSO-d_6_): δ 3.75 (s, 6H, 2x OCH_3_), 3.96 (s, 2H, CH_2_), 6.46 (t, *J* = 2.2 Hz, 1H, Ar-H-4), 6.51 (d, *J* = 2.2Hz, 2H, Ar-H-2,6) ppm. HRMS (ESI): m/z [M + H]^+^ calcd for C_10_H_11_NO_2_, 178.2165; found, 178.2158.

*2-(2,6-dibromophenyl)acetonitrile (8)*

Yield: 63 %; white crystals; mp 201-203 °C (lit[54] 208-209 °C). ^1^H NMR (400 MHz, DMSO-d_6_): δ 4.22 (s, 2H, CH_2_), 7.28 (t, *J* = 8.0 Hz, 1H, Ar-H-4), 7.78 (d, *J* = 8.0 Hz, 2H, Ar-H-3,5) ppm. HRMS (ESI): m/z [M + H]^+^ calcd for C_8_H_6_NBr_2_, 273.8867; found, 273.8864

*2-(3,5-dibromophenyl)acetonitrile (9)*

Yield: 58 %; white crystals; mp 63-65 °C (lit[55] 67-73 °C).^1^H NMR (400 MHz, DMSO-d_6_): δ 3.54 (s, 2H, CH_2_), 7.28 (d, *J* = 1.8 Hz, 2H, Ar-H-2,6), 7.41 (t, *J* = 1.8 Hz ,1H, Ar-H-4) ppm. HRMS (ESI): m/z [M + H]^+^ calcd for C_8_H_6_NBr_2_, 273.8859; found, 273.8862.

2-(3,5-dichlorophenyl)acetonitrile (10)

Yield: 77 %; white crystals; mp 68-71 °C (lit[56] 64-65 °C).^1^H NMR (400 MHz, DMSO-d_6_): δ 3.43 (s, 2H, CH_2_), 7.32 (d, *J* = 1.9 Hz, 2H, Ar-H-2,6), 7.49 (t, *J* = 1.9 Hz ,1H, Ar-H-4) ppm. HRMS (ESI): m/z [M + H]^+^ calcd for C_8_H_6_NCl_2_, 187.0537; found, 187.0543.

**Synthesis of Compounds 11, 13, 18, 20, 22, 24, 26, 31, 33, and 35**

NaH (0.029 g, 1.17 mmol) was added with stirring to 10 ml of 2-ethoxyethanol at 5 °C over a period of 10 minutes. The reaction mixture was allowed to warm to room temperature and then phenylacetonitrile (3.08 mmol) was added, followed by aldehyde **2** (0.4 g, 2.93 mmol). The reaction mixture was refluxed (ca. 130 °C) for 4h, then allowed to cool to room temperature and poured into cold water. After standing in refrigerator for 1h, the suspension was filtered and the precipitate washed with water, CH_3_CN and Et_2_O.

**Synthesis of Compounds 12, 14, 19, 21, 23, 25, 27, 32, 34 and 36**

To a suspension of diamine (0.65 mmol) in DMF, NaH (0.018 g, 0.75 mmol) was added and the mixture was stirred at room temperature for 1h. *tert*-Butyl isocyanate (0.083 ml, 0.70 mmol) was added and the reaction mixture was stirred overnight at room temperature. The solvent was evaporated under reduced pressure, water was added to the residue and the forming precipitate filtered and purified with flash column chromatography using ethyl acetate/chloroform (1/1) as eluent.

**Synthesis of Compounds 15 and 28**

A mixture of diamine (1.65 mmol), sulfamic acid (0.320 g, 3.30 mmol) and 4-(3-aminopropyl)morpholine (20 ml) was heated at 150 °C for 42 h. After cooling, the reaction mixture was suspended in water (30 ml), made alkaline with a saturated solution of aqueous NaHCO_3_ and extracted with CH_2_Cl_2_ (3 x 30 ml). The combined organic layers were washed with brine, dried over Na_2_SO_4_, filtered and evaporated under reduced pressure, giving a residue that was purified by column chromatography using chloroform/methanol (9/1) as eluent.

**Synthesis of Compounds 16 and 29**

To a solution of pyridopyrimidine **15** and **28** (0.37 mmol) in DMF, NaH (0.009 g, 0.37 mmol) was added and the mixture was stirred at room temperature for 1h. *tert*-Butyl isocyanate (0.044 ml, 0.37 mmol) was added and the reaction mixture stirred overnight at room temperature. The reaction mixture was concentrated in vacuo and the residue partitioned between EtOAc and water. The aqueous layer was extracted three times with EtOAc (3 x 30 ml) and the combined organic layers were washed with brine, dried over Na_2_SO_4_, filtered and evaporated under reduced pressure, giving a residue that was purified by column chromatography using ethyl acetate/methanol (4/1) as eluent.

**Synthesis of Compounds 17 and 30**

A mixture of diamine (1.00 mmol), sulfamic acid (0.194 g, 2.00 mmol) and 3-(dimethylamino)propylamine (10 ml) was refluxed (120 °C) for 72 h. The solvent was evaporated under reduced pressure and the residue partitioned between water and Et_2_O. The aqueous phase was extracted with Et_2_O (3 x 20 ml) and the combined organic layers washed with brine, dried over Na_2_SO_4_, filtered and evaporated under reduced pressure, giving a residue that was purified by column chromatography using chloroform/methanol (9/1) as eluent.

*6-phenylpyrido[2,3-d]pyrimidine-2,7-diamine (11)*

Yield: 79 %; pale brown crystals; mp >300 °C (lit[57] > 300°C). ^1^H NMR (400 MHz, DMSO-d_6_): δ 6.52 (br s, 2H, NCNH_2_N), 6.64 (br s, 2H, NCNH_2_C), 7.34-7.55 (m, 5H, Ar-H'), 7.64 (s, 1H, Ar-H), 8.66 (s, 1H, Ar-H) ppm. HRMS (ESI): m/z [M + H]^+^ calcd for C_13_H_12_N_5_, 238.1093; found, 238.1099. HPLC t_R_ = 9.137 min (100.00% at 220 nm, 100.00% at 254 nm).

*1-(2-amino-6-phenylpyrido[2,3-d]pyrimidin-7-yl)-3-tert-butylurea (12)*

Yield: 81 %; pale yellow crystals; mp > 300 °C. ^1^H NMR (400 MHz, DMSO-d_6_): δ 1.39 (s, 9H, C(CH_3_)_3_), 6.99 (br s, 1H, NHCO) 7.15 (br s, 2H, NCNH_2_N), 7.44-7.64 (m, 5H, Ar-H'), 7.99 (s, 1H, Ar-H), 8.94 (s, 1H, Ar-H), 10.06 (s, 1H, CONH) ppm. ^13^C NMR (DMSO-d_6_): δ 28.54, 57.45, 110.81, 118.65, 127.21, 128.75, 130.01, 136.24, 136.83, 148.42, 151.51, 157.24, 160.24, 163.58 ppm. HRMS (ESI): m/z [M + H]^+^ calcd for C_18_H_21_N_6_O, 337.1777; found, 337.1762. HPLC t_R_ = 14.677 min (100.00% at 220 nm, 100.00% at 254 nm).

*6-(3,5-dimethoxyphenyl)pyrido[2,3-d]pyrimidine-2,7-diamine (13)*

Yield: 82 %; off-white crystals; mp 297-300 °C (lit.[58] > 250 °C). ^1^H NMR (400 MHz, DMSO-d_6_): δ 3.79 (s, 6H, 2x OCH_3_), 6.38-6.82 (m, 7H, NCNH_2_N + NCNH_2_C + Ar-2',4',6'), 7.67 (s, 1H, Ar-H), 8.65 (s, 1H, Ar-H) ppm. ^13^C NMR (DMSO-d_6_): δ 55.63, 99.81, 104.03, 110.43, 118.73, 135.47, 138.25, 155.56, 157.21, 159.95, 163.76 ppm. HRMS (ESI): m/z [M + H]^+^ calcd for C_15_H_16_N_5_O_2_, 298.1304; found, 298.1292. HPLC t_R_ = 10.224 min (96.42 % at 220 nm, 98.60 % at 254 nm).

*1-(2-amino-6-(3,5-dimethoxyphenyl)pyrido[2,3-d]pyrimidin-7-yl)-3-tert-butylurea (14)*

Yield: 64 %; pale yellow crystals; mp > 300 °C (lit.[58] > 250 °C). ^1^H NMR (400 MHz, DMSO-d_6_): δ 1.39 (s, 9H, C(CH_3_)_3_), 3.81 (s, 6H, 2x OCH_3_), 6.63 (br s, 3H, NHCONH + NCNH_2_N), 7.10 (s, 1H, Ar-H-4'), 7.16 (s, 2H, Ar-H-2',6'), 8.00 (s, 1H, Ar-H), 8.93 (s, 1H, Ar-H), 10.04 (s, 1H, NHCONH) ppm. ^13^C NMR (DMSO-d_6_): δ 28.54, 55.81, 58.48, 100.45. 105.68, 109.32, 113.95, 119.03, 135.47, 136.12, 138.97, 151.68, 155.87, 158.78, 159.50, 160.72 ppm. HRMS (ESI): m/z [M + H]^+^ calcd for C_20_H_25_N_6_O_3_, 397.1988; found, 397.1981. HPLC t_R_ = 15.490 min (97.66 % at 220 nm, 99.30 % at 254 nm).

*6-(3,5-dimethoxyphenyl)-N2-(3-morpholinopropyl)pyrido[2,3-d]pyrimidine-2,7-diamine (15)*

Yield: 38 %; off-white crystals; mp 146-148 °C. ^1^H NMR (400 MHz, DMSO-d_6_): δ 1.65-1.80 (m, 2H, NCH_2_CH_2_CH_2_NH), 2.28-2.43 (m, 6H, NCH_2_CH_2_CH_2_NH + (CH_2_)_2_N), 3.30-3.38 (m, 2H, NCH_2_CH_2_CH_2_NH), 3.51-3.65 (m, 4H, (CH_2_)_2_O), 3.79 (s, 6H, 2x OCH_3_), 6.55 (m, 5H, NCNH_2_C + Ar-H-2',4',6'), 7.31 (br s, 1H, NCNHN), 7.67 (s, 1H, Ar-H), 8.64 (s, 1H, Ar-H) ppm. ^13^C NMR (DMSO-d_6_): δ 27.24, 40.31, 53.37, 55.23, 56.16, 66.19, 99.87, 106.54, 110.42, 118.39, 136.40, 139.04, 148.12, 156.54, 157.39, 159.97, 160.72 ppm. HRMS (ESI): m/z [M + H]^+^ calcd for C_22_H_29_N_6_O_3_, 425.2301; found, 425.2289. HPLC t_R_ = 10.805 min (99.29% at 220 nm, 99.89% at 254 nm).

*1-tert-butyl-3-(6-(3,5-dimethoxyphenyl)-2-(3-morpholinopropylamino)pyrido[2,3-d]pyrimidin-7-yl)urea (16)*

Yield: 46 %; off-white crystals; mp 81-85 °C. ^1^H NMR (400 MHz, DMSO-d_6_): δ 1.39 (s, 9H, C(CH_3_)_3_), 1.68-1.83 (m, 2H, NCH_2_CH_2_CH_2_NH), 2.28-2.42 (m, 6H, NCH_2_CH_2_CH_2_NH + (CH_2_)_2_NCH_2_), 3.38-3.48 (m, 2H, NCH_2_CH_2_CH_2_NH), 3.51-3.65 (m, 4H, (CH_2_)_2_O), 3.81 (s, 6H, 2x OCH_3_), 6.63 (br s, 3H, NHCONH + NCNH_2_N), 7.14 (s, 1H, Ar-H-4'), 7,19 (s, 2H, Ar-H-2',6'), 7.82 (br s, 1H, NCNHN), 8.00 (s, 1H, Ar-H), 8.91 (s, 1H, Ar-H), 10.40 (s, 1H, NHCONH) ppm. ^13^C NMR (DMSO-d_6_): δ 25.53, 28.67, 50.04, 53.39, 55.46, 56.22, 66.22, 100.29, 107.12, 109.19, 137.01, 138.01, 151.94, 154.47, 157.72, 160.91, 161.42 ppm. HRMS (ESI): m/z [M + H]^+^ calcd for C_27_H_38_N_7_O_4_, 524.2985; found, 524.2981. HPLC t_R_ = 14.747 min (98.76 % at 220 nm, 99.47 % at 254 nm).

*6-(3,5-dimethoxyphenyl)-N2-(3-(dimethylamino)propyl)pyrido[2,3-d]pyrimidine-2,7-diamine (17)*

Yield: 24 %; pale yellow crystals; mp 92-95 °C. ^1^H NMR (400 MHz, DMSO-d_6_): δ 1.61-1.75 (m, 2H, NCH_2_CH_2_CH_2_NH), 2.13 (s, 6H, CH_3_)_2_N), 2.27 (t, 2H, *J* = 6.8 Hz, NCH_2_CH_2_CH_2_NH), 3.34-3.47 (m, 2H, NCH_2_CH_2_CH_2_NH), 3.78 (s, 6H, 2x OCH_3_), 6. 52 (br s, 2H, NCNH_2_C), 6.55 (s, 1H, Ar-H-4'), 6.58 (s, 1H, Ar-H-2',6'), 7.29 (br s, 1H, NCNHN), 7.67 (s, 1H, Ar-H), 8.64 (s, 1H, Ar-H) ppm. ^13^C NMR (DMSO-d_6_): δ 27.65, 40.11, 45.27, 55.23, 57.06, 99.87, 106.54, 110.68, 118.37, 136.40, 139.05, 148.29, 156.24, 157.93, 159.96, 160.72 ppm. HRMS (ESI): m/z [M + H]^+^ calcd for C_20_H_27_N_6_O_2_, 383.2195; found, 383.2191. HPLC t_R_ = 11.837 min (96.35% at 220 nm, 97.57% at 254 nm).

*6-(3,5-bis(trifluoromethyl)phenyl)pyrido[2,3-d]pyrimidine-2,7-diamine (18)*

Yield: 75 %; off-white crystals; mp > 300 °C. ^1^H NMR (400 MHz, DMSO-d_6_): δ 6.78 (br s, 2H, NCNH_2_N), 6.86 (br s, 2H, NCNH_2_C), 7.81 (s, 1H, Ar-H), 8.11 (s, 3H, *J* = 8.1 Hz, Ar-H-2',4',6'), 8.68 (s, 1H, Ar-H) ppm. ^13^C NMR (DMSO-d_6_): δ 108.55, 117.62, 121.26, 122.01, 124.72, 129.80, 130.56, 130.89, 138.11, 139.86, 160.67, 161.09, 163.82 ppm. HRMS (ESI): m/z [M + H]^+^ calcd for C_15_H_10_N_5_F_6_, 374.0840; found, 374.0847. HPLC t_R_ = 14.953 min (97.68% at 220 nm, 98.95% at 254 nm).

*1-(2-amino-6-(3,5-bis(trifluoromethyl)phenyl)pyrido[2,3-d]pyrimidin-7-yl)-3-tert-butylurea (19)*

Yield: 39 %; white crystals; mp > 300 °C. ^1^H NMR (400 MHz, DMSO-d_6_): δ 1.37 (s, 9H, C(CH_3_)_3_), 7.23 (br s, 2H, NCNH_2_N), 8.08 (s, 1H, NHCO), 8.14 (s, 2H, Ar-H-2',6'), 8.15 (s, 1H, Ar-H-4'), 8.32 (s, 1H, Ar-H), 8.93 (s, 1H, Ar-H), 9.98 (s, 1H, CONH) ppm. ^13^C NMR (DMSO-d_6_): δ 28.80, 49.90, 109.29, 119.25, 121.66, 124.72, 130.54, 138.91, 139.80, 152.51, 155.18, 158.45, 162.02, 164.05 ppm. HRMS (ESI): m/z [M + H]^+^ calcd for C_20_H_18_N_6_F_6_O, 473.1525; found, 473.1526. HPLC t_R_ = 18.719 min (98.57% at 220 nm, 99.08% at 254 nm).

*6-(3,5-difluorophenyl)pyrido[2,3-d]pyrimidine-2,7-diamine (20)*

Yield: 66%; pale brown crystals; mp > 300 °C. ^1^H NMR (400 MHz, DMSO-d_6_): δ 6.75 (br s, 4H, NCNH_2_N + NCNH_2_C), 7.18 (d, 2H, *J =* 6.0 Hz, Ar-H-2'6'), 7.23-7.30 (m, 1H, Ar-H-4'), 7.73 (s, 1H, Ar-H), 8.65 (s, 1H, Ar-H) ppm. ^13^C NMR (DMSO-d_6_): 103.08, 108.39, 112.25, 118.26, 137.22, 140.66, 148.43, 157.58, 160.52, 163.75, 164.36 ppm. HRMS (ESI): m/z [M + H]^+^ calcd for C_13_H_10_N_5_F_2_, 274.0904; found, 274.0909. HPLC t_R_ = 9.991 min (95.52% at 220 nm, 97.13% at 254 nm).

*1-(2-amino-6-(3,5-difluorophenyl)pyrido[2,3-d]pyrimidin-7-yl)-3-tert-butylurea (21)*

Yield: 52 %; white crystals; mp > 300 °C. ^1^H NMR (400 MHz, DMSO-d_6_): δ 1.45 (s, 9H, C(CH_3_)_3_), 7.29 (br s, 2H, NCNH_2_N), 7.33 (d, 2H, *J =* 6.0 Hz, Ar-H-2',6'), 7.37-7.46 (m, 1H, Ar-H-4'), 7.73 (s, 1H, NHCO), 8.09 (s, 1H, Ar-H), 8.98 (s, 1H, Ar-H), 10.09 (s, 1H, CONH) ppm. ^13^C NMR (DMSO-d_6_): δ 28.82, 50.01, 103.88, 109.18, 112.69, 112.91, 119.37, 139.02, 152.12, 154.75, 158.29, 162.02, 164.05 ppm. HRMS (ESI): m/z [M + H]^+^ calcd for C_18_H_19_N_6_F_2_O, 373.1588; found, 373.1579. HPLC t_R_ = 15.776 min (97.60% at 220 nm, 98.77% at 254 nm).

*6-(3,5-dichlorophenyl)pyrido[2,3-d]pyrimidine-2,7-diamine (22)*

Yield: 66%; white crystals; mp > 300 °C. ^1^H NMR (400 MHz, DMSO-d_6_): δ 6.75 (br s, 2H, NCNH_2_N), 6.78 (br s, 2H, NCNH_2_C), 7.49 (d, 2H, *J =* 1.9 Hz, Ar-H-2',6'), 7.63 (t, 1H, *J =* 1.9 Hz, Ar-H-4'), 7.72 (s, 1H, Ar-H), 8.65 (s, 1H, Ar-H) ppm. ^13^C NMR (DMSO-d_6_): δ 108.46, 117.83, 127.17, 127.68, 134.37, 137.47, 140.74, 160.55, 160.57, 160.95, 163.76 ppm. HRMS (ESI): m/z [M + H]^+^ calcd for C_13_H_10_N_5_Cl_2_, 306.0313; found, 306.0313. HPLC t_R_ = 13.547 min (97.21% at 220 nm, 98.27% at 254 nm).

*1-(2-amino-6-(3,5-dichlorophenyl)pyrido[2,3-d]pyrimidin-7-yl)-3-tert-butylurea (23)*

Yield: 68 %; white crystals; mp 179-182 °C. ^1^H NMR (400 MHz, DMSO-d_6_): δ 1.44 (s, 9H, C(CH_3_)_3_), 7.28 (br s, 2H, NCNH_2_N), 7.60 (d, 2H, *J =* 6.2 Hz, Ar-H-2',6'), 7.73-7.80 (m, 1H, Ar-H-4'), 7.92 (s, 1H, NHCO), 8.08 (s, 1H, Ar-H), 8.96 (s, 1H, Ar-H), 10.08 (s, 1H, CONH) ppm. ^13^C NMR (DMSO-d_6_): δ 28.83, 49.98, 109.23, 119.03, 127.81, 128.94, 134.55, 139.36, 152.26, 154.80, 158.30, 161.99, 164.03 ppm. HRMS (ESI): m/z [M + H]^+^ calcd for C_18_H_19_N_6_OCl_2_, 405.0997; found, 405.0978. HPLC t_R_ = 18.164 min (98.88% at 220 nm, 99.34% at 254 nm).

*6-(2,6-difluorophenyl)pyrido[2,3-d]pyrimidine-2,7-diamine (24)*

Yield: 80 %; pale brown crystals; mp > 300 °C. ^1^H NMR (400 MHz, DMSO-d_6_): δ 6.70 (br s, 2H, NCNH_2_N), 6.76 (br s, 2H, NCNH_2_C), 7.17-7.27 (m, 2H, Ar-H-3',5'), 7.47-7.58 (m, 1H, Ar-H-4'), 7.73 (s, 1H, Ar-H), 8.64 (s, 1H, Ar-H) ppm. ^13^C NMR (DMSO-d_6_): δ 109.87, 110.21, 117.75, 118.53, 129.17, 137.58, 148.25, 156.13, 157.94, 160.43, 163.65 ppm. HRMS (ESI): m/z [M + H]^+^ calcd for C_13_H_10_N_5_F_2_, 274.0904; found, 274.0902. HPLC t_R_ = 8.867 min (99.56% at 220 nm, 99.82% at 254 nm).

*1-(2-amino-6-(2,6-difluorophenyl)pyrido[2,3-d]pyrimidin-7-yl)-3-tert-butylurea (25)*

Yield: 63 %; off-white crystals; mp > 300 °C. ^1^H NMR (400 MHz, DMSO-d_6_): δ 1.39 (s, 9H, C(CH_3_)_3_), 7.23-7.33 (m, 4H, NCNH_2_N + Ar-H-3',5'), 7.54-7.64 (m, 1H, Ar-H-4'), 7.37-7.46 (m, 1H, Ar-H-4'), 8.03 (s, 1H, NHCO), 8.09 (s, 1H, Ar-H), 8.93 (s, 1H, Ar-H), 10.04 (s, 1H, CONH) ppm. ^13^C NMR (DMSO-d_6_): δ 28.86, 50.02, 109.56, 110.18, 118.32, 118.91, 129.37, 136.53, 148.32, 151.04, 157.29, 159.86, 160.73, 163.74 ppm. HRMS (ESI): m/z [M + H]^+^ calcd for C_18_H_19_N_6_F_2_O, 373.1588; found, 373.1575. HPLC t_R_ = 14.603 min (96.24% at 220 nm, 96.99% at 254 nm).

*6-(2,6-dichlorophenyl)pyrido[2,3-d]pyrimidine-2,7-diamine (26)*

Yield: 78 %; off-white crystals; mp > 300 °C (lit.[58] 336-338 °C)  ^1^H NMR (400 MHz, DMSO-d_6_): δ 6.61 (br s, 2H, NCNH_2_N), 6.75 (br s, 2H, NCNH_2_C), 7.46 (dd, *J* = 7.3, 1.6 Hz, 1H, Ar-H-4'), 7.57-7.65 (m, 3H, Ar-H-3'5' + Ar-H), 8.64 (s, 1H, Ar-H) ppm. ^13^C NMR (DMSO-d_6_): δ 111.54, 118.52, 126.42, 130.32, 133.57, 135.73, 137.32, 148.63, 155.72, 157.91, 163.27 ppm. HRMS (ESI): m/z [M + H]^+^ calcd for C_13_H_10_N_5_Cl_2_, 306.0313; found, 306.0305. HPLC t_R_ = 10.630 min (95.36 % at 220 nm, 96.76 % at 254 nm).

*1-(2-amino-6-(2,6-dichlorophenyl)pyrido[2,3-d]pyrimidin-7-yl)-3-tert-butylurea (27)*

Yield: 35 %; off-white crystals; mp > 300 °C. ^1^H NMR (400 MHz, DMSO-d_6_): δ 1.39 (s, 9H, C(CH_3_)_3_), 7.28 (br s, 2H, NCNH_2_N), 6.99 (dd, *J* = 7.6, 1.6 Hz, Ar-H-4'), 7.62-7.68 (m, 2H, Ar-H-3',6'), 7.73 (s, 1H, NHCO), 7.98 (s, 1H, Ar-H), 8.93 (s, 1H, Ar-H), 10.18 (s, 1H, CONH) ppm. ^13^C NMR (DMSO-d_6_): δ 28.86, 55.52, 110.56, 118.32, 127.43, 129.97, 133.62, 135.21, 136.85, 148.21, 151.04, 157.86, 160.62, 163.56 ppm. HRMS (ESI): m/z [M + H]^+^ calcd for C_18_H_19_N_6_OCl_2_, 405.0997; found, 405.0989. HPLC t_R_ = 15.649 min (97.30% at 220 nm, 99.27% at 254 nm).

*6-(2,6-dichlorophenyl)-N2-(3-morpholinopropyl)pyrido[2,3-d]pyrimidine-2,7-diamine (28)*

Yield: 45 %; white crystals; mp 124-127 °C. ^1^H NMR (400 MHz, DMSO-d_6_): δ 1.69-1.78 (m, 2H, NCH_2_CH_2_CH_2_NH), 2.29-2.44 (m, 6H, NCH_2_CH_2_CH_2_NH + (CH_2_)_2_N), 3.30-3.38 (m, 2H, NCH_2_CH_2_CH_2_NH), 3.52-3.63 (m, 4H, (CH_2_)_2_O), 6.55 (br s, 2H, NCNH_2_C), 7.31 (br s, 1H, NCNHN), 7.42-7.52 (m, 1H, Ar-H-4'), 7.56-7.63 (m, 3H, Ar-H-3',5' + Ar-H), 8.63 (s, 1H, Ar-H) ppm. ^13^C NMR (DMSO-d_6_): δ 27.35, 40.92, 55.85, 60.76, 66.26, 110.42, 118.39, 170.42, 130.36, 133.65, 135.84, 136.74, 148.58, 156.52, 157.26, 161.27 ppm. HRMS (ESI): m/z [M + H]^+^ calcd for C_20_H_23_N_6_OCl_2_, 433.1337; found, 433.1316. HPLC t_R_ = 12.335 min (98.27% at 220 nm, 99.33% at 254 nm).

*1-tert-butyl-3-(6-(2,6-dichlorophenyl)-2-(3-morpholinopropylamino)pyrido[2,3-d]pyrimidin-7-yl)urea (29)*

Yield: 56 %; white crystals; mp 232-234 °C (lit.[58] 236-240 °C). ^1^H NMR (400 MHz, DMSO-d_6_): δ 1.39 (s, 9H, C(CH_3_)_3_), 1.67-1.86 (m, 2H, NCH_2_CH_2_CH_2_NH), 2.30-2.41 (m, 6H, NCH_2_CH_2_CH_2_NH + (CH_2_)_2_N), 3.35-3.48 (m, 2H, NCH_2_CH_2_CH_2_NH + CH_2_CH_3_ ), 3.53-3.62 (m, 4H, (CH_2_)_2_O), 7.53 (dd, *J* = 7.0, 2.0 Hz, 1H, Ar-H-4'), 7.60-7.68 (m, 2H, Ar-H-3'5'), 7.74 (br s, 1H, NCNHN), 7.86 (s, 1H, NHCO), 7.95 (s, 1H, Ar-H), 8.90 (s, 1H, Ar-H), 10.47 (s, 1H, CONH) ppm. ^13^C NMR (DMSO-d_6_): δ 27.25, 28.97, 40.68, 52.63, 57.51, 62.22, 66.84, 110.29, 118.42, 127.16, 130.72, 133.73, 135.96, 137.27, 148.01, 151.69, 157.35, 160.74, 161.95 ppm. HRMS (ESI): m/z [M + H]^+^ calcd for C_25_H_32_N_7_O_2_Cl_2_, 532.2021; found, 532.2000. HPLC t_R_ = 16.966 min (100.00% at 220 nm, 100.00% at 254 nm).

*6-(2,6-dichlorophenyl)-N2-(3-(dimethylamino)propyl)pyrido[2,3-d]pyrimidine-2,7-diamine (30)*

Yield: 24 %; off-white crystals; mp 241-244 °C. ^1^H NMR (400 MHz, DMSO-d_6_): δ 1.63-1.77 (m, 2H, NCH_2_CH_2_CH_2_NH), 2.14 (s, 6H, CH_3_)_2_N), 2.28 (t, 2H, *J* = 7.0 Hz, NCH_2_CH_2_CH_2_NH), 3.33-3.46 (m, 2H, NCH_2_CH_2_CH_2_NH), 6.55 (br s, 2H, NCNH_2_C), 7.29 (br s, 1H, NCNHN), 7.41-7.52 (m, 1H, Ar-H-4'), 7.54-7.65 (m, 3H, Ar-H-3',5' + Ar-H), 8.63 (s, 1H, Ar-H) ppm. ^13^C NMR (DMSO-d_6_): δ 27.79, 40.26, 47.54, 55.74, 110.87, 118.74, 127.64, 130.53, 133.47, 135.78, 137.82, 148.75, 156.47, 157.47, 161.87 ppm. HRMS (ESI): m/z [M + H]^+^ calcd for C_18_H_21_N_6_Cl_2_, 391.1205; found, 391.1198. HPLC t_R_ = 12.207 min (95.23% at 220 nm, 96.77% at 254 nm).

*6-(2-chloro-6-fluorophenyl)pyrido[2,3-d]pyrimidine-2,7-diamine (31)*

Yield: 45 %; pale brown crystals; mp > 300 °C. ^1^H NMR (400 MHz, DMSO-d_6_): δ 6.64 (br s, 2H, NCNH_2_N), 6.76 (br s, 2H, NCNH_2_C), 7.34 (t, 1H, Ar-H-4'), 7.41-7.57 (m, 2H, Ar-H-3',5'), 7.66 (s, 1H, Ar-H), 8.65 (s, 1H, Ar-H) ppm. ^13^C NMR (DMSO-d_6_): δ 107.94, 111.34, 114.81, 123.72, 125.73, 131.02, 134.82, 138.36, 148.21, 156.26, 157.53, 159.39, 163.85 ppm. HRMS (ESI): m/z [M + H]^+^ calcd for C_13_H_10_N_5_ClF, 290.0609; found, 290.0606. HPLC t_R_ = 9.653 min (96.56% at 220 nm, 96.99% at 254 nm).

*1-(2-amino-6-(2-chloro-6-fluorophenyl)pyrido[2,3-d]pyrimidin-7-yl)-3-tert-butylurea (32)*

Yield: 54 %; off-white crystals; mp > 300 °C. ^1^H NMR (400 MHz, DMSO-d_6_): δ 1.39 (s, 9H, C(CH_3_)_3_), 7.29 (s, 2H, NCNH_2_N), 7.39 (t, 1H, Ar-H-4'), 7.50-7.61 (m, 2H, Ar-H-3',5'), 7.89 (s, 1H, NHCO), 8.04 (s, 1H, Ar-H), 8.93 (s, 1H, Ar-H), 10.11 (s, 1H, CONH) ppm. ^13^C NMR (DMSO-d_6_): δ 28.34, 49.59, 110.65, 112.91, 118.31, 123.45, 125.21, 131.45, 134.26, 137.34, 148.76, 151.87, 156.43, 159.29, 160.78, 163.88 ppm. HRMS (ESI): m/z [M + H]^+^ calcd for C_18_H_19_N_6_OClF, 389.1293; found, 389.1293. HPLC t_R_ = 15.190 min (97.57% at 220 nm, 98.50% at 254 nm).

*6-(2,6-dibromophenyl)pyrido[2,3-d]pyrimidine-2,7-diamine (33)*

Yield: 85 %; pale yellow crystals; mp 285-289 °C (lit.[58] 284 °C). ^1^H NMR (400 MHz, DMSO-d_6_): δ 6.56 (br s, 2H, NCNH_2_N), 6.73 (br s, 2H, NCNH_2_C), 7.30 (t, *J* = 8.0 Hz, 1H, Ar-H-4'), 7.54 (s, 1H, Ar-H), 7.79 (d, 2H, Ar-H-3'5'), 8.65 (s, 1H, Ar-H) ppm. ^13^C NMR (DMSO-d_6_): δ 111.54, 118.52, 122.05, 131.56, 133.57, 135.42, 136.19, 143.27, 148.35, 156.17, 157.74, 163.68 ppm. HRMS (ESI): m/z [M + H]^+^ calcd for C_13_H_10_N_5_Br_2_, 393.9303; found, 393.9294. HPLC t_R_ = 11.130 min (96.64 % at 220 nm, 97.89 % at 254 nm).

*1-(2-amino-6-(2,6-dibromophenyl)pyrido[2,3-d]pyrimidin-7-yl)-3-tert-butylurea (34)*

Yield: 65 %; white crystals; mp > 300 °C (lit.[58] > 300 °C). ^1^H NMR (400 MHz, DMSO-d_6_): δ 1.39 (s, 9H, C(CH_3_)_3_), 7.29 (br s, 2H, NCNH_2_N), 7.38 (t, *J* = 8.2 Hz, 1H, Ar-H-4'), 7.49 (s, 1H, NHCONH), 7.85 (d, *J* = 8.2 Hz, 2H, Ar-H-3'5'), 7.94 (s, 1H, Ar-H), 8.95 (s, 1H, Ar-H), 10.18 (s, 1H, NHCONH) ppm. ^13^C NMR (DMSO-d_6_): δ 28.80, 50.07, 109.02, 120.49, 125.68, 132.41, 132.52, 135.64, 139.72, 152.18, 154.40, 158.43, 161.94, 164.17 ppm. HRMS (ESI): m/z [M + H]^+^ calcd for C_18_H_19_N_6_OBr_2_, 492.9987; found, 492.9985. HPLC t_R_ = 15.867 min (97.37 % at 220 nm, 98.69 % at 254 nm).

*6-(benzo[d][1,3]dioxol-5-yl)pyrido[2,3-d]pyrimidine-2,7-diamine (35)*

Yield: 74 %; brown crystals; mp > 300 °C. ^1^H NMR (400 MHz, DMSO-d_6_): δ 6.08 (s, 2H, CH_2_), 6.57 (br s, 2H, NCNH_2_N), 6.65 (br s, 2H, NCNH_2_C), 6.90 (dd, *J* = 8.1, 1.8 Hz, 1H, Ar-H-2'), 6.97-7.05 (m, 2H, Ar-H-3',6'), 7.59 (s, 1H, Ar-H), 8.63 (s, 1H, Ar-H) ppm. ^13^C NMR (DMSO-d_6_): δ 101.13, 108.64, 109.23, 120.36, 122.31, 130.76, 136.19, 146.83, 147.59, 160.09, 161.18, 163.51 ppm. HRMS (ESI): m/z [M + H]^+^ calcd for C_14_H_12_N_5_O_2_, 282.0991; found, 282.0987. HPLC t_R_ = 9.697 min (97.70% at 220 nm, 98.79% at 254 nm).

*1-(2-amino-6-(benzo[d][1,3]dioxol-5-yl)pyrido[2,3-d]pyrimidin-7-yl)-3-tert-butylurea (41)*

Yield: 52 %; off-white crystals; mp > 300 °C. ^1^H NMR (400 MHz, DMSO-d_6_): δ 1.39 (s, 9H, C(CH_3_)_3_), 6.13 (s, 2H, CH_2_), 6.95 (dd, *J* = 8.1, 1.8 Hz, 1H, Ar-H-2'), 7.04-7.14 (m, 3H, NHCO + Ar-H-3',6'), 7.17 (br s, 2H, NCNH_2_N), 7.94 (s, 1H, Ar-H), 8.92 (s, 1H, Ar-H), 10.08 (s, 1H, CONH) ppm. ^13^C NMR (DMSO-d_6_): δ 28.97, 50.12, 101.56, 107.98, 108.89, 120.57, 122.78, 129.86, 136.35, 146.26, 148.31, 151.32, 160.09, 161.18, 163.51 ppm. HRMS (ESI): m/z [M + H]^+^ calcd for C_19_H_21_N_6_O_3_, 381.1675; found, 381.1666. HPLC t_R_ = 15.062 min (98.11% at 220 nm, 99.60% at 254 nm).

**References**

47. Huber W (1943) 2,4-Diamino-5-(4-methyl-5-β-hydroxyethylthiazolium chloride)-methylpyrimidine Hydrochloride, a New Analog of Thiamin. J Am Chem Soc 65: 2222–2226.

48. Bag S, Tawari NR, Degani MS, Queener SF (2010) Design, synthesis, biological evaluation and computational investigation of novel inhibitors of dihydrofolate reductase of opportunistic pathogens. Bioorg Med Chem 18: 3187–3197.

49. Hwang Seok-Ho, Moorefield CN, Hee-Chuil C, Wang P, Newkome GR (2006) Synthesis of 5-substituted 1,3-[bis(2,2′:6′,2″-terpyridin-4′-ylethynyl)]benzene ligands and their coordination-driven self-assembly. Designed Monomers & Polymers: 413–424.

50. Wheeler HL, Clapp SH (n.d.) Researches on Halogen Amino Acids: 3,5-Dibromphenylalanine, IV. American Chemical Journal 1908: 337–348.

51. Asinger F, Lock G (1933) Über den 3, 5-Dichlor-benzaldehyd. Monatshefte für Chemie 62: 344–348.

52. Tashiro M, Nakayama K (1984) Preparation of bromobenzoic acids from the corresponding bromotoluenes via the Krohnke method. Org Prep Proced Int 16: 379–383.

53. Haller HL, Schaffer PS (1939) Action of isobutylmagnesium bromide on 3,4,5-trimethoxybenzonitrile. J Am Chem Soc: 2175–2177.

54. Ugi I, Fetzer U, Eholzer U, Knupfer H, Offermann K (1965) Neuere Methoden der präparativen organischen Chemie IV. Isonitril‐Synthesen. Angewandte Chemie 77: 492–504.

55. Chiba S, Zhang L, Ang GY, Hui BW-Q (2010) Generation of Iminyl Copper Species from α-Azido Carbonyl Compounds and Their Catalytic C−C Bond Cleavage under an Oxygen Atmosphere. Org Lett 12: 2052–2055.

56. Liskey CW, Liao X, Hartwig JF (2010) Cyanation of Arenes via Iridium-Catalyzed Borylation. J Am Chem Soc 132: 11389–11391.

57. Hamby JM, Connolly CJ, Schroeder MC, Winters RT, Showalter HD, et al. (1997) Structure-activity relationships for a novel series of pyrido[2,3-d]pyrimidine tyrosine kinase inhibitors. J Med Chem 40: 2296–2303.

58. Schroeder MC, Hamby JM, Connolly CJ, Grohar PJ, Winters RT, et al. (2001) Soluble 2-substituted aminopyrido[2,3-d]pyrimidin-7-yl ureas. Structure-activity relationships against selected tyrosine kinases and exploration of in vitro and in vivo anticancer activity. J Med Chem 44: 1915–1926.
