# Supplementary material for: 6-Arylpyrido[2,3-d]pyrimidines as Novel ATP-Competitive Inhibitors of Bacterial D-Alanine:D-Alanine Ligase
Source: PLoS One. 2012 Aug 2;7(8):e39922. doi: 10.1371/journal.pone.0039922 (PMC3410885; doi:10.1371/journal.pone.0039922)
Supplement: Information S2 — Isothermal titration calorimetry. (DOCX) [file pone.0039922.s005.docx]

**Isothermal titration calorimetry**

**Fitting of ITC titration curve**

Binding of the investigated DdlB inhibitors into the DdlB binding site is relatively weak therefore the stoichiometry of the investigated process cannot be determined from ITC titration curve. That is why we decided rather not to fit the number of binding sites, N, during the data analysis but to propose the single binding site model for fitting of the model equation to the experimental data points. According to the one-binding site model, binding of a ligand to a protein can be described as


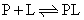
 (1)

where P is the protein (DdlB), L is the ligand (DdlB inhibitor) which binds to the protein binding site, and PL is the 1:1 protein-ligand complex. The equilibrium between the species can be expressed by an apparent equilibrium constant, $K_{\text{b}}$,

$K_{\text{b}}=\frac{\left[\text{PL}\right]}{\left[\text{P}\right]\left[\text{L}\right]}$ (2)

where $\left[\text{PL}\right]$is an equilibrium concentration of protein-ligand complex, $\left[\text{P}\right]$is an equilibrium concentration of free protein, and $\left[\text{L}\right]$is an equilibrium concentration of free ligand in the titration cell. Taking into account the mass balance equations for total concentration of protein, $\left[\text{P}\right]_{\text{tot}}$, and ligand, $\left[\text{L}\right]_{\text{tot}}$, in the titration cell

$\begin{matrix} \left[\text{P}\right]_{\text{tot}}=\left[\text{P}\right]+\left[\text{PL}\right] \\ \left[\text{L}\right]_{\text{tot}}=\left[\text{L}\right]+\left[\text{PL}\right] \end{matrix}$ (3)

a ratio $\nu=\frac{\left[\text{PL}\right]}{\left[\text{P}\right]_{\text{tot}}}$ can be expressed with use of a ratio $r=\frac{\left[\text{L}\right]_{\text{tot}}}{\left[\text{P}\right]_{\text{tot}}}$ and the equilibrium constant as

$\nu=\frac{K_{\text{b}}\left[\text{P}\right]_{\text{tot}}\left(r-\nu\right)}{1+K_{\text{b}}\left[\text{P}\right]_{\text{tot}}\left(r-\nu\right)}$. (4)

Furthermore, the quadratic equation for $\nu$ is being resolved

$\nu=\frac{1}{2}\left(1+r+\frac{1}{K_{\text{b}}\left[\text{P}\right]_{\text{tot}}}-\sqrt{\left(1+r+\frac{1}{K_{\text{b}}\left[\text{P}\right]_{\text{tot}}}\right)^{2}-4r}\right)$. (5)

Enthalpy of solution in the titration cell after i-th injection, $H_{\text{i}}$, can be expressed as

$H_{\text{i}} = n_{1}\bar{H}_{1} + n_{\text{P}}\bar{H}_{\text{P}} + n_{\text{L}}\bar{H}_{\text{L}} + n_{\text{PL}}\bar{H}_{\text{PL}}=n_{1}\bar{H}_{1} + n_{\text{P}}^{0}\bar{H}_{\text{P}} + n_{2}\bar{H}_{\text{L}} + n_{\text{PL}}\left(\bar{H}_{\text{PL}}-\bar{H}_{\text{P}}-\bar{H}_{\text{L}}\right)$ (6)

where $n_{1}$,$n_{\text{P}}$,$n_{\text{L}}$, and $n_{\text{PL}}$ represent the number of moles of solvent, protein, ligand, and protein-ligand complex, and $\bar{H}_{1}$,$\bar{H}_{\text{P}}$,$\bar{H}_{\text{L}}$, and $\bar{H}_{\text{PL}}$ are the corresponding partial molar enthalpies. $n_{\text{P}}^{0}$ and $n_{2}$ represent the total number of moles of protein and ligand in the titration cell, respectively. By taking a partial derivative of the enthalpy with respect to $n_{2}$ at constant *p*, *T*, and $n_{1}$ and taking into account the Gibbs-Duhem equation one can obtain

$\bar{H}_{2}=\left(\frac{\partial H_{\text{i}}}{\partial n_{2}}\right)_{p,T,n_{1}} =\bar{H}_{\text{L}}+\left(\frac{\partial n_{\text{PL}}}{\partial n_{2}}\right)\text{Δ}H_{\text{b}}$ (7)

where $\text{Δ}H_{\text{b}}=\bar{H}_{\text{PL}}-\bar{H}_{\text{P}}-\bar{H}_{\text{L}}$.

During the ITC experiment enthalpy changes accompanying ligand addition into the calorimeter cell, $\text{Δ}H_{\text{exp}}$, are measured. After subtracting the blank titration ($n_{\text{P}}^{0}=0$),$\text{Δ}H_{\text{blank}}$, the obtained relative partial molar enthalpy can be expressed as $\text{Δ}H=\text{Δ}H_{\text{exp}}-\text{Δ}H_{\text{blank}}=\bar{H}_{2}-\bar{H}_{\text{L}}$.[14] Combining this relation with the Eq. (7) and assuming that the corresponding $\bar{H}_{\text{L}}$ values in sample and blank solutions are the same and $\text{Δ}H_{\text{b}}$is concentration independent and thus equal to its value in standard state ($\text{Δ}H_{\text{b}}=\text{Δ}H_{\text{b}}^{0}$), one obtains

$\text{Δ}H=\text{Δ}H_{\text{b}}^{0}\left(\frac{\partial n_{\text{PL}}}{\partial n_{2}}\right)=\text{Δ}H_{\text{b}}^{0}\left(\frac{\partial\nu}{\partial r}\right)$. (8)

Even more, after calculating the above partial derivative, $\left(\frac{\partial\nu}{\partial r}\right)$, the measured $\text{Δ}H$can be expressed in terms of two variables: $K_{\text{b}}$ (or $\text{Δ}G_{\text{b}}^{0}$) and $\text{Δ}H_{\text{b}}^{0}$ (be aware that all these variables are temperature dependent)

$\text{Δ}H=\frac{1}{2}\text{Δ}H_{\text{b}}^{0}\left(1-\frac{r-1+\frac{1}{K_{\text{b}}\left[\text{P}\right]_{\text{tot}}}}{\sqrt{\left(1+r+\frac{1}{K_{\text{b}}\left[\text{P}\right]_{\text{tot}}}\right)^{2}-4r}}\right)$. (9)

The values for both parameters were obtained by fitting of the model equation to the experimental data points. The model equation was compared to the experimental curve via the χ^2^ function defined as

$\chi^{2} = \sum_{i} \left(\frac{\text{Δ}H_{\text{i}}^{\text{exp}} - \text{Δ}H_{\text{i}}^{\text{calc}}}{\text{Δ}\left(\text{Δ}H_{\text{i}}\right)}\right)^{2}$ (10)

where $\text{Δ}H_{\text{i}}^{\text{exp}}$ and $\text{Δ}H_{\text{i}}^{\text{calc}}$ represent the experimental and model enthalpy, whereas $\text{Δ}\left(\text{Δ}H_{\text{i}}\right)$ is the absolute error in measured enthalpy. Best-fit values of the above-mentioned parameters were calculated by χ^2^ function minimization using the Levenberg-Marquardt nonlinear regression algorithm.[59] These values were further used to determine the corresponding binding Gibbs free energy,$\text{Δ}G_{\text{b}}^{0}$, and entropy,$\text{Δ}S_{\text{b}}^{0}$, at 37°C.

**References**

59. Lah J, Maier NM, Lindner W, Vesnaver G (2001) Thermodynamics of Binding of (R)- and (S)-Dinitrobenzoyl Leucine to Cinchona Alkaloids and Their tert-Butylcarbamate Derivatives in Methanol:  Evaluation of Enantioselectivity by Spectroscopic (CD, UV) and Microcalorimetric (ITC) Titrations. J Phys Chem B 105: 1670–1678.
